# Low doses of bioherbicide favour prion aggregation and propagation *in vivo*

**DOI:** 10.1038/s41598-018-25966-9

**Published:** 2018-05-23

**Authors:** Pierre-André Lafon, Thibaut Imberdis, Yunyun Wang, Joan Torrent, Mike Robitzer, Elisabeth Huetter, Maria-Teresa Alvarez-Martinez, Nathalie Chevallier, Laurent Givalois, Catherine Desrumaux, Jianfeng Liu, Véronique Perrier

**Affiliations:** 1 MMDN, University Montpellier, EPHE, INSERM, U1198- EiAlz team, PSL Research University, Montpellier, F-34095 France; 2 Cellular Signaling Laboratory, College of Life Science and Technology, Huazhong University of Science and Technology, Wuhan, Hubei China; 3 Institut Charles Gerhardt Montpellier, UMR 5253 CNRS-ENSCM-UM, Matériaux Avancés pour la Catalyse et la Santé, Montpellier, France; 4 Etablissement Confiné d’Expérimentation A3/L3, CECEMA, US009 Biocampus, UMS 3426, Université Montpellier, Montpellier, F-34095 France; 5 Present Address: Ann Romney Center for Neurologic Diseases, Brigham and Women’s hospital, Harvard Medical School, Boston, MA USA

## Abstract

Public concerns over the use of synthetic pesticides are growing since many studies have shown their impact on human health. A new environmental movement in occidental countries promoting an organic agriculture favours the rebirth of botanical pesticides. These products confer an effective alternative to chemical pesticides such as glyphosate. Among the biopesticides, the α-terthienyls found in the roots of *Tagetes* species, are powerful broad-spectrum pesticides. We found that an α-terthienyl analogue with herbicidal properties, called A6, triggers resistant SDS oligomers of the pathogenic prion protein PrP^Sc^ (rSDS-PrP^Sc^) in cells. Our main question is to determine if we can induce those rSDS-PrP^Sc^ oligomers *in vitro* and *in vivo*, and their impact on prion aggregation and propagation. Using wild-type mice challenged with prions, we showed that A6 accelerates or slows down prion disease depending on the concentration used. At 5 mg/kg, A6 is worsening the pathology with a faster accumulation of PrP^Sc^, reminiscent to soluble toxic rSDS-PrP^Sc^ oligomers. In contrast, at 10 and 20 mg/kg of A6, prion disease occurred later, with less PrP^Sc^ deposits and with rSDS-PrP^Sc^ oligomers in the brain reminiscent to non-toxic aggregates. Our results are bringing new openings regarding the impact of biopesticides in prion and prion-like diseases.

## Introduction

With the increase of life expectancy in the developed countries, the prevalence of age-related disorders continues to rise. The neurodegenerative disease epidemic raises major concerns, among them, the absence of efficient treatments to cure these disorders and the prohibited costs for societies. Thus, reinforcing the prevention and a better identification of the exogenous environmental risk factors involved in these pathologies could also be a way to slow down the epidemic and limit the economic burden for occidental countries^1^.

Retrospectively, the 20^th^ century was marked by the tremendous development of heavy and fine petrochemical industries that led to an annual production of 500 million tons of chemical derivatives, among them 140 million tons of fertilizers and about 5 million tons of pesticides^2,3^. Fertilizers and pesticides were extensively used to develop industrial farming and agro-industry leading to their release into all environmental media (water, soil, air). In consequence, numerous pesticide residues were introduced into the food chain raising many concerns regarding their impact on human health^1,3,4^. The role of pesticides in the occurrence of neurodegenerative diseases is pointed-out since residues of pesticides were detected in human tissues and biological fluids, even decades after their use was prohibited, such as the organochlorine pesticide, dichlorodiphenyltrichloroethane (DDT)^1^. Recently, it was demonstrated that dichlorodiphenyldichloroethylene (DDE) levels, a metabolite of the DDT banned in 1972, were almost 4 fold-higher in the serum of Alzheimer’s disease (AD) patients with ApoE ε4 allele, than in control subjects^5^. Another category of pesticides, paraquat and rotenone, were suppressed from the European market in 2007 and 2008^6,7^, respectively. These compounds were incriminated since several epidemiological studies in humans showed an increased incidence of Parkinson’s disease (PD) in agricultural workers who were exposed to high doses of these pesticides, as well as population living in contaminated rural environment^8,9^. Experimental studies aiming to mimic this contamination showed that the administration of rotenone is able to trigger a Parkinsonian’s syndrome in rodent animal models^10^. Regarding prion diseases such as Bovine Spongiform Encephalopathy (BSE), the role of phosmet, an anti-parasitis used to decontaminate cows before their slaughtering, was suspected to have a role in the mad-cow disease epidemic^11^. Experiments in cell culture showed that the normal prion protein, PrP^C^, was more expressed at the cell surface in the presence of phosmet. Thus, it was suggested that PrP^C^ was more prone to interact with the prion misfolded isoform PrP^Sc^ and initiate the transconformation process^12^.

It has been shown that neurological disorders as diverse as AD, PD and Creutzfeldt-Jacob disease (CJD) share a common pathogenic mechanism involving the aggregation and deposition of misfolded proteins in the central nervous system. Although the type of aggregated proteins is disease-specific (Aβ peptides, α-synuclein or PrP^Sc^), they all share a “prion-like” mechanism of cell-to-cell propagation^13^. These aggregation pathways involve toxic oligomeric species leading to fibril formation and amyloid deposition^14^. Our hypothesis is that pesticides could modify the balance between the different species (native proteins, misfolded proteins, oligomers and fibrils) by shifting the equilibrium towards misfolded proteins, oligomers and/or aggregates. We previously showed, in a drug screening assay on prion-infected cells, that a compound, called A6 is a strong inducer of SDS-resistant oligomers of the pathogenic form of the prion protein PrP^Sc^ (rSDS-PrP^Sc^)^15,16^. Moreover, this compound was previously described for its herbicidal activity^17^ and is structurally related to the family of α-terthienyls (α-Ter), natural molecules synthesized by plants such as marigolds and Asteraceae^18^. These plants were traditionally used by gardeners as ≪home made≫ preparations for pest control^18–20^. α-Ter and plant-derived insecticides are now the matter of a new expanding market because of the environmental movement in favour of organic agriculture that allows some botanical pest control. In addition, in January 2015, the French National Assembly voted a law (0’Phyto)^21^ that prohibits the use of chemical pesticides in public and private gardens starting 2017. So we can expect a raise in the use of biopesticides whose effects on human health are largely unknown.

Since α-Ter biopesticide analogue A6 can trigger rSDS-PrP^Sc^ oligomers, our main question was to determine if we could induce *in vivo* those rSDS-PrP^Sc^ oligomers and what could be their impact on prion aggregation and propagation. Can this compound worsen or not the pathology? Indeed, many oligomeric species have been described in the literature: soluble oligomers were described as the most neurotoxic species in neurodegenerative diseases (AD, PD, prions)^22,23^, whereas some oligomeric species, described as amorphous aggregates, are not able to replicate and propagate prion diseases^24–26^. Using wild-type mice challenged with prions, we showed that A6 can accelerate or slow-down prion disease depending on the concentration used. At 5 mg/kg, the prion pathology occurred earlier in mice and is associated with a faster accumulation of PrP^Sc^ deposits in brain tissue sections compared to control group. By contrast, at 10 and 20 mg/kg, prion disease occurred later in treated mice and is associated with a slower accumulation of PrP^Sc^ deposits in brain tissue sections compared to control groups. *In vitro* experiments performed on prion-infected brain homogenates to understand the mechanism of action showed that above a threshold of 1 mM, A6 induces a strong precipitation of PrP^Sc^, with appearance of insoluble rSDS-PrP^Sc^ oligomers in the pellets. However, at 0.25 mM of A6, rSDS-PrP^Sc^ oligomers were detected in the supernatant suggesting that at lower concentrations, A6 rather promotes soluble toxic species. Our results showed that the α-Ter biopesticide analogue A6 has opposite effects in a wild-type murine model of prion diseases. *In vitro* and *in vivo* exposures to low doses of A6 are likely more prone to induce soluble toxic species leading to shorter survival life in the exposed animals. These results are bringing new openings regarding the potential impact of biopesticides in prion and prion-like diseases.

## Results

### The biopesticide analogue A6 interacts with PrP fibrils

**Figure 1:**
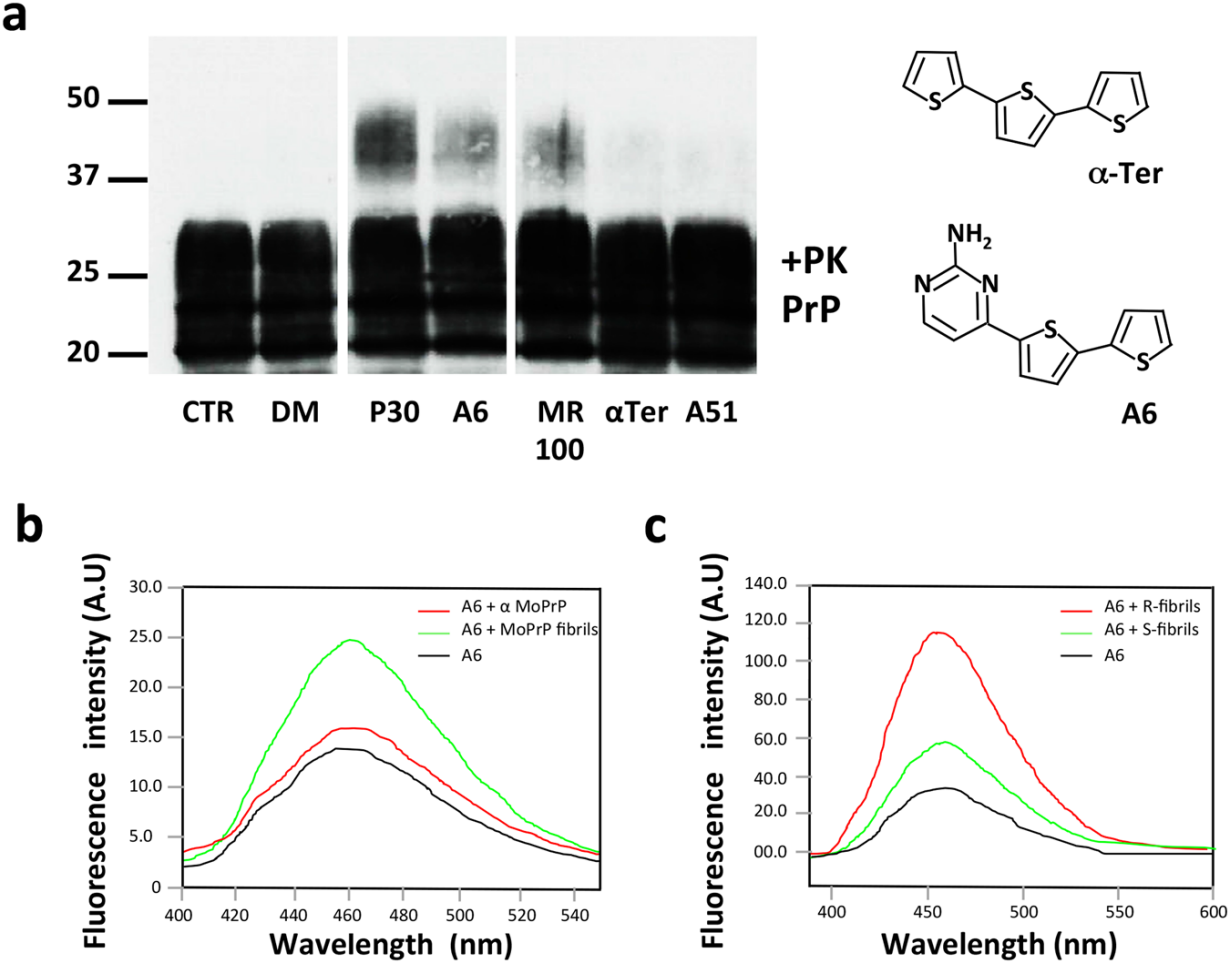
A6 promotes PK rSDS-PrP^Sc^ oligomers and interacts with PrP fibrils. (**a**) Comparison of several compounds for their ability to induce PK rSDS-PrP^Sc^ oligomers. Prion-infected N2a58/22L cellular lysates were incubated with 0.5 mM of P30, A6, MR100, α-Ter and A51 for 1 h. Samples were then PK digested at 37 °C for 1 h. Immunoblot was probed with SAF mix antibodies (mixture of three monoclonal anti-PrP antibodies: SAF60, SAF69 and SAF70) for prion detection. Molecular weight markers are indicated on the left side of the immunoblot. The cropped blot is used in this figure and the full-length blot is presented in Supplementary Figure S6. Chemical structures of A6 and α-Ter, 2 compounds described for their herbicidal properties. **(b)** Fluorescence interaction studies between A6 compound and PrP. Purified full-length recombinant mouse PrP (MoPrP23-230) protein, at 4.4 μM, either soluble or fibrillar, were incubated with 50 μM of A6 compound in 1% DMSO, 50 mM MES pH 6, during 2 h at 25 °C. Emission spectra were recorded between 400 and 550 nm by exciting at λ_ex_ = 372 nm: 50 μM of A6 (black), 50 μM of A6 + α-soluble MoPrP23-230 (red) and 50 μM of A6 + fibrils of MoPrP23-230 (green). **(c)** Interaction studies of A6 compound with hamster PrP fibrils. Hamster-S or -R fibrils at a concentration of 4.4 μM were incubated with 40 μM of A6 compound in 1% DMSO, 50 mM MES pH 6, during 2 h at room temperature. Fluorescence spectra were recorded between 400 and 600 nm: 40 μM of A6 (black), 40 μM of A6 + S-fibrils (green) and 40 μM of A6 + R-fibrils (red).

Previously, we identified from a cellular drug screening on prion-infected cells a family of thienyl pyrimidine compounds allowing us to detect proteinase K (PK) rSDS-PrP^Sc^ oligomers by immunoblotting^15^. Because A6 is an analogue of the compound α-Ter, and was also described for its herbicidal properties, our aim was to determine if α-Ter also exhibit an oligomer-inducing activity on prion-infected cells. In a fast comparison assay, we have incubated prion-infected cellular lysates with various compounds for 1 hour. Then after PK digestion, samples were analysed on immunoblot. The results showed that P30, one of the lead compounds identified is able to induce a strong signal of PK rSDS-PrP^Sc^ oligomers, as well as A6 and MR100, although in a lesser extend due to their ability to precipitate prions in the tubes. However, α-Ter is not able to induce PK rSDS-PrP^Sc^ oligomers from cellular lysates, nor has the ability to precipitate prions (Fig. 1a). Thus we decided to focus our study only on A6 compound and further explore the impact of rSDS-PrP^Sc^ oligomers on prion propagation.

*In vitro* interaction studies such as Surface Plasmon Resonance Studies (SPR or Biacore) to calculate binding affinities between A6 and PrP, were difficult to perform due to A6 insolubility in aqueous buffer^15,26^. However, since α-Ter, the plant molecule from which A6 is derived was originally isolated on its blue-fluorescent properties^27^, we thus examined if A6 compound could exhibit the same property. The aim was to perform interaction studies based on A6 intrinsic fluorescence. A broad peak of absorption from 320 nm to 500 nm was found upon spectral analyses as previously described^28^. After excitation at 372 nm, we observed a fluorescent signal with a maximal emission at 460 nm, which is nearly identical to the one of α-Ter (λ_ex_ = 361 nm and λ_em_ = 428 nm) (Fig. 1b,c). Taking advantage of these physical properties, we performed interaction studies by following the fluorescence of A6 in presence or in absence of recombinant mouse PrP23–230 proteins, either soluble, folded in α-helices (α-MoPrP) or MoPrP fibrils (Fig. 1b). We observed a minor modification of fluorescence intensity after 2 hours of incubation of A6 with α-MoPrP, compared to the spectrum of A6 alone. By contrast, in presence of PrP fibrils, the maximal fluorescence signal at 460 nm is doubled compared to the control A6 alone. The fluorescence of A6 is strongly enhanced in the presence of PrP fibrils illustrating the binding of A6 with PrP fibrils but not with α-MoPrP (Fig. 1b). These results are consistent with our previous studies showing the specificity of P30 compound, one of the thienyl pyrimidine family members, towards PrP^Sc^ and not PrP^C ^^15,16^. We also performed fluorescence binding experiments using another model of fibrils, Hamster S- or R-fibrils produced by different agitation modes (shaking or rotated), and displaying distinctive morphologies (R-fibrils are curved, S-fibrils are straight) linked to different folding patterns of cross-β-structures (Fig. 1c)^29,30^. Our aim was to see whether A6 could preferentially bind to hamster S- or R- fibrils. Using the fluorescent property of A6 as a tracer, we showed that the compound interacts with both types of fibrils although the intensity of the fluorescence is higher for R-fibrils (about 5 fold) than S-fibrils (about 2 fold) by comparison to the A6 spectrum alone (Fig. 1c).

### Treatment of prion-inoculated mice with 5 mg/kg of A6 decreased their survival time with a faster accumulation of PrP^Sc^ in their brains

**Figure 2:**
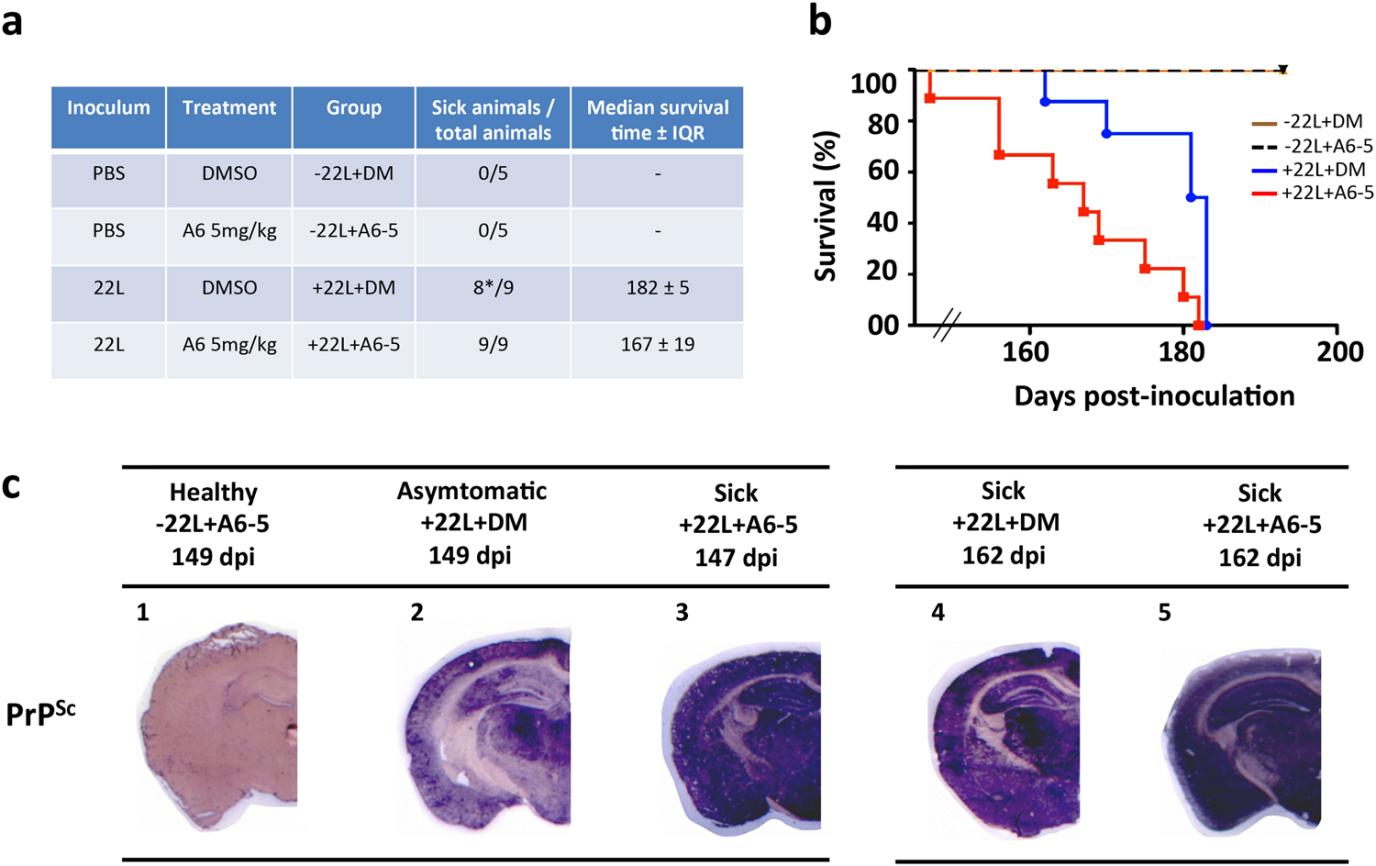
Mice treated with 5 mg/kg of A6 compound exhibit a decreased lifespan and an increased PrP^Sc^ burden. (**a**) Table summarizing the different groups of animals: prion inoculations, treatment performed, number of sick animals out of total number of animals (*means that in this group, one mouse was sacrificed while asymptomatic) and median survival time ± IQR (interquartile range). **(b)** Kaplan-Meier survival curves of mice intra-cerebrally (i.c.) inoculated with 5 μL of 22L prions, and treated with 50 μL of DMSO (n = 9, blue) or with 5 mg/kg of A6 (n = 9, red) by intraperitoneal (i.p.) route, (+22L + DM versus +22L + A6–5: non–parametric Mantel-Cox log-rank test, ** *p-*value = 0,0067). Healthy control mice, inoculated with 5 μL of PBS, were treated with 5 mg/kg of A6 (n = 5, black dashed, −22L + A6–5) or non-treated (n = 5, brown, −22L + DM). **(c)** Left panel: PET-blots analysis of coronal sections of control healthy mice non-inoculated with prions and treated with 5 mg/kg of A6 (−22L + A6–5) **(1)**; mice inoculated with 22L prions and either treated with DMSO (+22L + DM) **(2)** or treated with A6 (+22L + A6–5) **(3)**. Mice were all sacrificed at the same time (149, 149 and 147 dpi respectively) either in an asymptomatic or sick stage. Right panel: PET-blots analysis of coronal sections of sick mice: +22L + DM **(4)** and +22L + A6–5 **(5)** sacrificed at the same time (162 and 163 dpi, respectively). Sections were labelled with SAF84 antibody to detect PrP^Sc^ and revealed with Vectastain ABC-AmP kit.

Because A6 interacts with recombinant PrP fibrils and has the ability to form PK rSDS-PrP^Sc^ oligomers in prion-infected cells, our objective was to determine if we could form those oligomers *in vivo* and if they could have an impact on prion propagation and animals’ survival. Groups of animals were inoculated with 22L prion strain into the striatum through stereotaxic surgery to have precision in the injection site and in the volume injected. Indeed, it has been showed that the median survival time of mice challenged with prions varies with the brain inoculation site^31^. Mice were then treated with 5 mg/kg of A6 or with an equivalent volume of the solvent alone (50 μL of pure DMSO) by intra-peritoneal (i.p.) route (Fig. 2a). The treatment started one week after mice infection, twice a week for 5 weeks. All the prion-infected animals developed the disease and were sacrificed at the terminal stage, while PBS-inoculated mice remained healthy during the whole experiment, whatever the treatment was, DMSO or A6 (Fig. 2a). As shown in Fig. 2b, the median survival time ± IQR (interquartile range) of prion-infected mice treated with 5 mg/kg of A6 is significantly shorter than the control group having received 50 μL of pure DMSO, 167 ± 19 and 182 ± 5 days post-injection (dpi), respectively (Fig. 2a,b). Statistical significant difference between curves was performed using Log-rank (Mantel-Cox) test based on median survival time (*p-*value = 0.0067 **, n = 18), represented in a box-and-whiskers graph expressed as median values with 10 and 90 percentile interval (Supplementary Fig. S1a). When the first mouse in the A6-treated group needed to be euthanized (147 dpi), we also sacrificed animals in the control groups: a DMSO-treated mouse and a PBS-inoculated mouse treated with 5 mg/kg of A6, nearly at the same time (149 dpi), even though they were without symptoms, in order to compare prion levels in their brains (Fig. 2c,1–3). Histological analyses using the PET-blot technique allow us to evaluate PrP^Sc^ deposits in brain tissue sections. It is rather a qualitative than quantitative method, allowing us to see the aggregation state in the brain between different groups at a given point. Our results showed that prion-inoculated mice treated with A6 has accumulated a substantial amount of PrP^Sc^ in the brain compared to the asymptomatic DMSO treated mouse, suggesting that the treatment with A6 induces a faster accumulation of PrP^Sc^ than in the control group. This result is consistent with the shorter median survival time observed for prion-infected mice treated with A6, suggesting that mice died earlier because they have accumulated PrP^Sc^ more quickly in their brains. As expected, the non-inoculated mice treated with A6 compound did not exhibit PrP^Sc^ deposits in their brains. Comparison of mice at the terminal stage of the disease (162–163 dpi) (Fig. 2c,4–5) showed that PrP^Sc^ deposits are equivalent in the brain tissue sections of the mice treated with A6 compared to DMSO-treated mice. To confirm that animals died of prion disease, histopathological analyses were done to check for spongiosis, by a Hematoxylin/Eosin (HE) staining (Supplementary Fig. S2a–d) and for astrogliosis, by GFAP immunolabelling (Supplementary Fig. S2e–h) on brain tissue sections of animals treated with DMSO or with A6 at 5 mg/kg. These two parameters are rather qualitative markers and informative of the pathological prion status of the animals.

**Figure 3:**
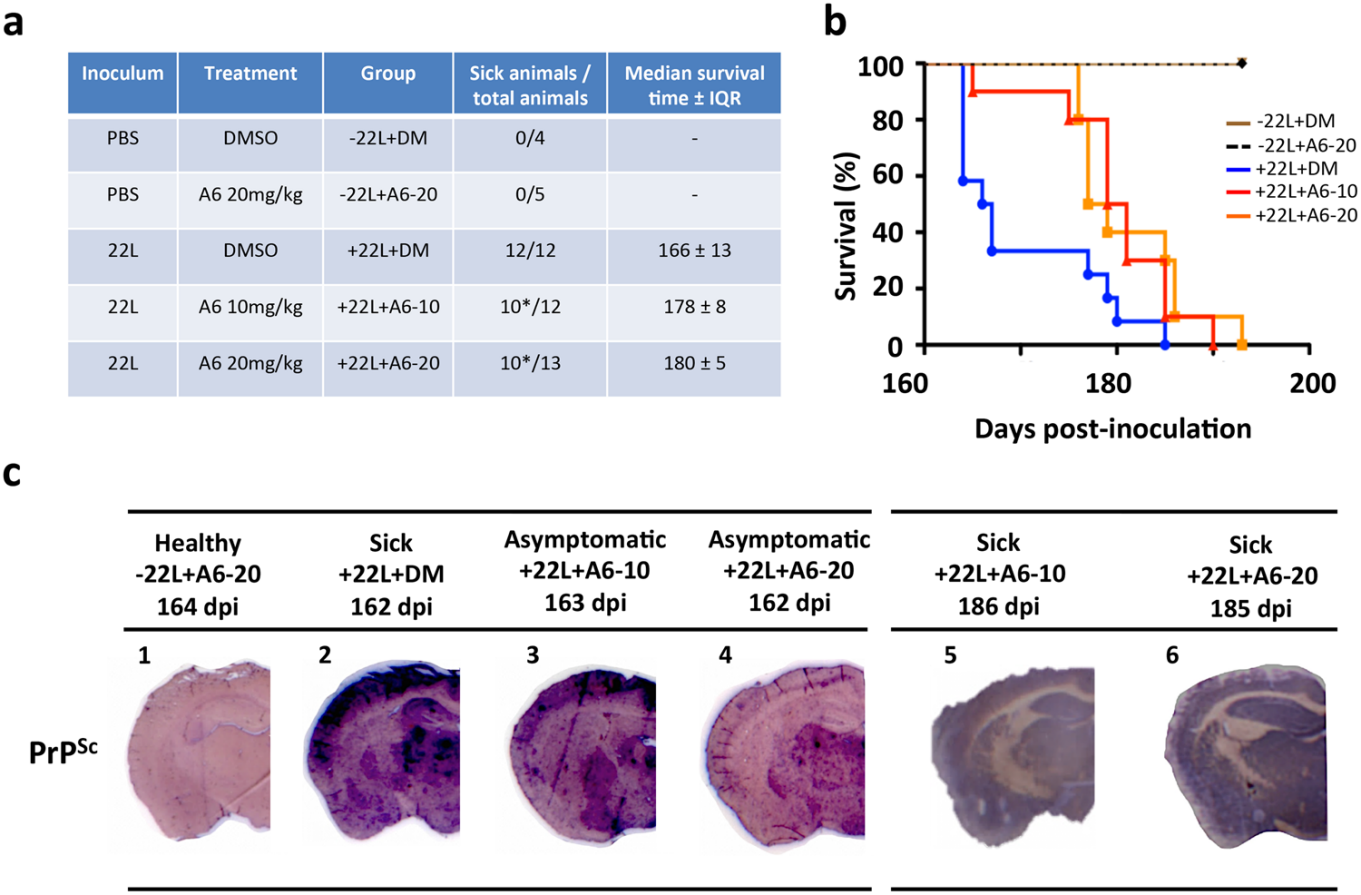
Mice treated with 10 and 20 mg/kg of A6 showed an increased survival time with a slower accumulation of PrP^Sc^ in their brains. (**a**) Table summarizing the different groups of animals (*means that in this group, 2–3 mice were sacrificed while asymptomatic). (**b**) Kaplan-Meier survival curves of mice i.c. inoculated with 5 μL of 22L prions, and treated i.p. either with 100 μL of DMSO (n = 12, blue), or with 10 mg/kg (n = 12, red) or 20 mg/kg of A6 (n = 13, orange) (+22L + DM versus +22L + A6–10: non–parametric Mantel-Cox log-rank test, *p-value = 0,012 and Wilcoxon test, **p-value = 0,0092. + 22L + DM versus +22L + A6–20: non-parametric Mantel-Cox log-rank test, *p-value = 0,016 and Wilcoxon test, **p-value = 0,0092). (**c**) Left panel: PET blot analysis of coronal tissue sections of healthy mice non-inoculated with prions and treated with 20 mg/kg of A6 (−22L + A6–20) (**1**); mice inoculated with prions and treated with equivalent volume of DMSO (+22L + DM) (**2**); mice inoculated with prions and treated either with 10 mg/kg of A6 (+22L + A6–10) (**3**), or 20 mg/kg of A6 (+22L + A6–20) (**4**). All mice were sacrificed at the same time either in an asymptomatic or sick status for comparison of amyloid load (164, 162, 163 and 162 dpi, respectively). Right panel: PET-blots analysis of sick mice: +22L + A6–10 (**5**) and +22L + A6–20 (**6**). Sections were labelled with SAF84 antibody to detect PrP^Sc^ and revealed with Vectastain ABC-AmP kit.

**Figure 4:**
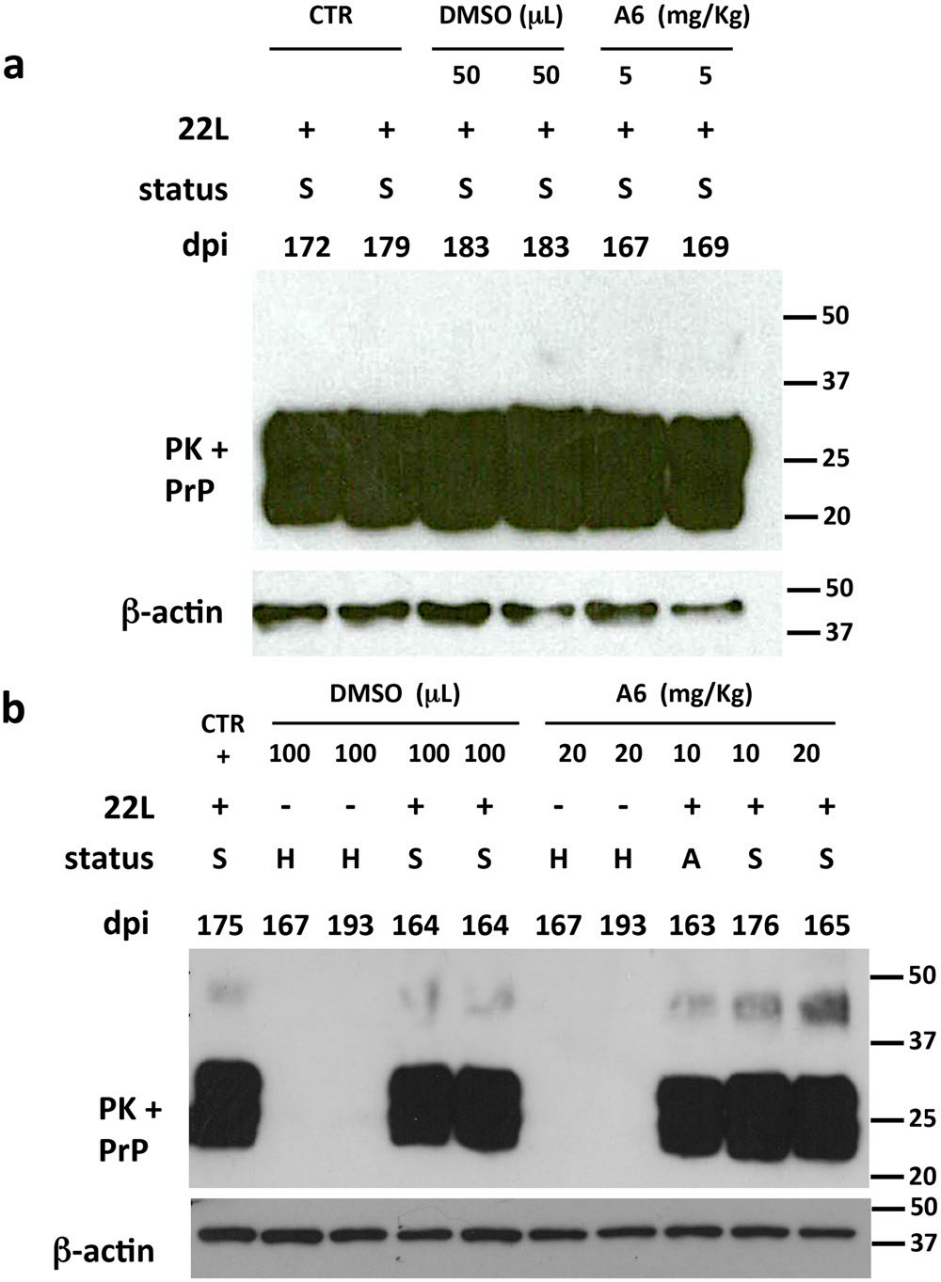
PK rSDS-PrP^Sc^ oligomers are not detected in the brain of mice treated with 5 mg/kg of A6 but are present in mice brains treated with 10 and 20 mg/kg of A6. (**a**) Brain homogenates from sick (S) mice inoculated with 22L prion strain (+) and either non-treated (CTR) or treated with DMSO (50 μL), or with 5 mg/kg of A6 compound, were PK-digested (PK+) at 37 °C for 1 h. Reaction was stopped by a cocktail of proteases inhibitor. Samples were analysed by immunoblotting using SAF mix antibodies able to detect PK rSDS-PrP^Sc^ oligomers. Numbers indicate the incubation time of animals (days post-inoculation, dpi) starting after prion infection. Protein loading controls were performed using an anti-β-actin antibody on each sample, taken before PK digestion. Molecular weight markers are indicated on the right side of immunoblots. (**b**) Brain homogenates from mice inoculated (+) or not (−) with the 22L prion strain, were either non-treated (CTR), or treated with DMSO (100 μL) or with 10 or 20 mg/kg of A6 compound, and were analysed by immunoblotting after PK digestion (PK+). General mice status at the time of the sacrifice is indicated by capital letters (S: sick, H: healthy and A: asymptomatic). Numbers indicate the incubation time of animals (days post-inoculation, dpi) starting after prion infection. Immunoblots were probed with SAF mix antibodies for prion detection or with anti-β-actin antibody, as protein loading controls. Molecular weight markers are indicated on the right side of immunoblots.

**Figure 5:**
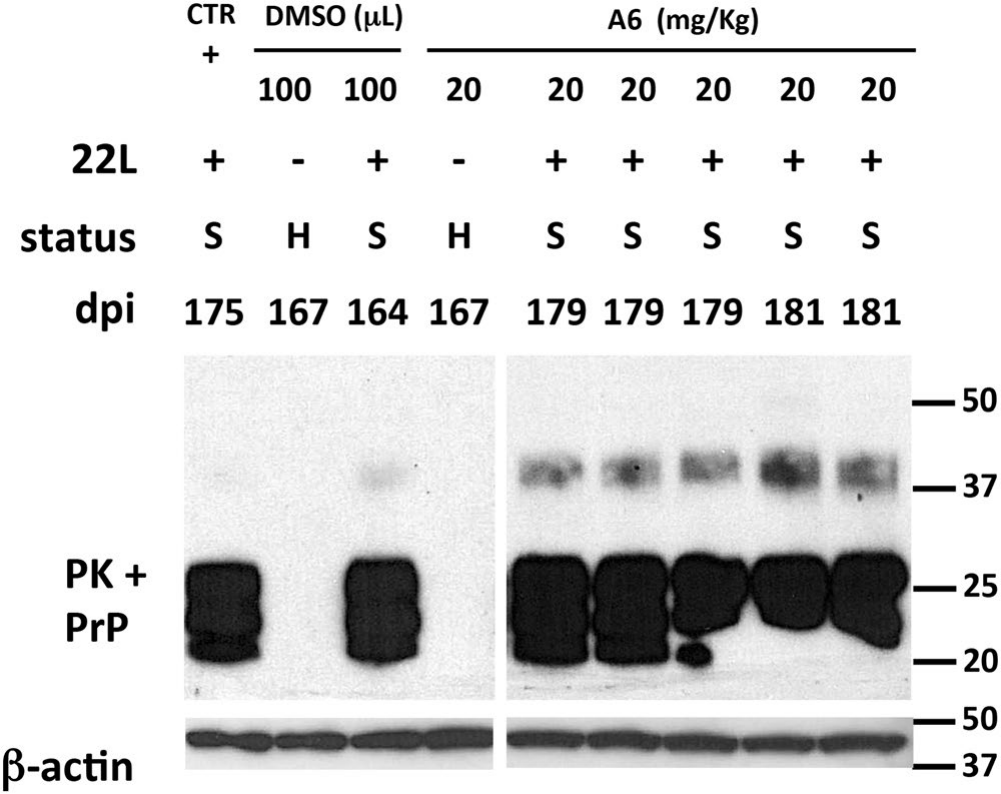
PK rSDS-PrP^Sc^ oligomers are present in all the brains of mice treated with 20 mg/kg of A6. Brain homogenates of mice non treated (CTR); treated with DMSO (100 μL) or treated with 20 mg/kg of A6 compound, challenged (+) or not (−) with the 22L prion strain, were PK-digested. Samples were analysed by western blot using SAF mix antibodies showing PK resistant oligomers of PrP^Sc^. Capital letters below each lane correspond to the general mice status at the time of sacrifice (S: sick; H: healthy). Numbers indicate the incubation time of animals (days post-inoculation, dpi) starting after prion infection. An anti-β-actin antibody was used as protein-loading controls, before PK digestion of samples. Molecular weight markers are indicated on the right side of each panel. The cropped blot is used in this figure and the full-length blot is presented in Supplementary Fig. S7.

### Treatment of prion-inoculated mice with 10 and 20 mg/kg of A6 increased the survival time of animals with a slower accumulation of PrP^Sc^ in their brains

Because the treatment of prion-infected mice with 5 mg/kg of A6 led to shorter survival time and faster accumulation of PrP^Sc^ deposits in mice, our aim was to evaluate the effect of higher concentrations of A6, as 10 and 20 mg/kg, to see if the treatment can worsen or not the pathology. As expected, all the mice inoculated with 22L prion strain developed the disease, whereas PBS-inoculated animals did not, either they were treated with pure DMSO (100 μL) or A6 at 20 mg/kg (Fig. 3a). Surprisingly, mice treated with 10 and 20 mg/kg of A6 presented an increase of their survival time compared to the DMSO-treated group (Fig. 3b). The median survival time ± IQR of prion-infected mice treated with 10 and 20 mg/kg of A6 are 178 ± 8 and 180 ± 5 dpi, respectively, which are significantly higher than the 166 ± 13 dpi of the DMSO-treated group (Fig. 3a,b). Statistical significant difference between curves was calculated using Log-rank (Mantel-Cox) test based on median survival time for A6 groups *versus* DMSO (*p-*values < 0.05 *n = 32) represented in a Box-and-whiskers graph expressed as median values with 10 and 90 percentile interval (Supplementary Fig. S1b). Thus, by increasing the concentration of A6 from 5 to 10 and 20 mg/kg, the effect observed on animal lifespan is opposite. As we previously did, when the first mouse in the DMSO-treated group needed to be euthanized (162 dpi), we also sacrificed animals at the same time in the A6-treated groups presenting no symptoms, as well as a healthy mouse non-inoculated with prions and treated with A6 at 20 mg/kg. In this way, we can evaluate prion levels in their brains at the same time and the impact of the A6 treatment at 10 and 20 mg/kg on prion propagation and aggregation. Histological analyses using PET-blot technique allow us to detect PrP^Sc^ deposits in animal brain tissues (Fig. 3c). Remarkably, we can see that the levels of PrP^Sc^ deposits are inversely proportional to the concentration of A6: the more A6 is concentrated, the less we observed PrP^Sc^ deposits in their brains (Fig. 3c,1–4). Our results suggest that the treatment with A6 slows down the rate of accumulation of PrP^Sc^ compared to the control group treated with DMSO. These results are consistent with the median survival times observed for prion-infected mice treated with A6 at 10 and 20 mg/kg, suggesting that mice die later, likely because they have accumulated PrP^Sc^ more slowly in their brains. Comparison of mice at the terminal stage of the disease (185 dpi) showed that PrP^Sc^ deposits are equivalent in brain tissue sections between the prion-inoculated groups (Fig. 3c,5–6). Histopathological analyses were also performed to confirm the presence of spongiosis (Supplementary Fig. S3a,1–6) and astrogliosis (Supplementary Fig. S3b,1–6) in brain tissue sections of animals. Results confirmed that all sick mice died of prion disease.

**Figure 6:**
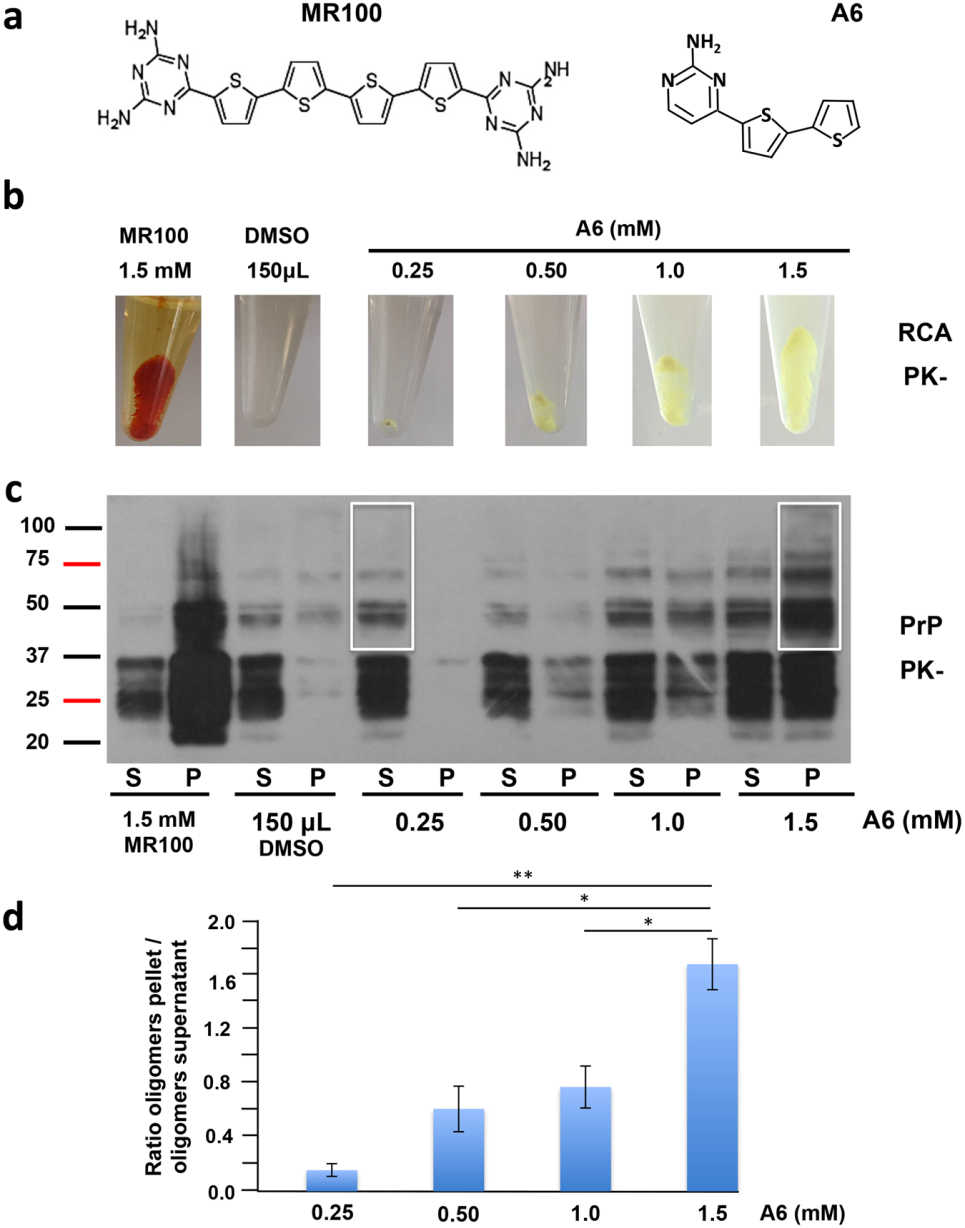
A6 induces *in vitro* soluble and/or insoluble rSDS-PrP^Sc^ oligomers in a dose dependent manner. **(a)** Chemical structures of MR100 and A6 compounds. **(b)** Representative images of yellow precipitates after Rapid Centrifugation Assay (RCA)^26^. Prion-infected brain samples (50 μL) were incubated with 1.5 mM of MR100 compound (positive control), or with an equivalent volume of DMSO (150 μL, negative control), or with a range of concentrations of A6 compound (from 0.25 mM to 1.5 mM) according to RCA protocol described previously and performed without PK digestion (PK-). After a centrifugation step, a visible precipitate appeared for MR100 (orange colour) and A6 (yellow colour) but not for DMSO sample. The size of the pellet is proportional to the concentration of A6. **(c)** Western blot analysis of the supernatants and pellets after samples were processed by RCA. Immunoblot was probed with SAF mix antibodies to detect the presence of rSDS-PrP^Sc^. Molecular weight markers are indicated on the left side of the panel. **(d)** Histogram representing the ratio of the levels of oligomers in the pellet versus oligomers in the supernatant for each concentration of A6. One-way ANOVA (**p-value = 0.0086) followed by Tukey’s multiple comparison test was performed for statistical significance (*p < 0.05, **p < 0.01).

### Mice treated with 5 mg/kg of A6 did not revealed PK SDS-resistant PrP^Sc^ oligomers in their brains

We then analysed brain homogenates of animals treated with A6 at 5 mg/kg to see if they contain some PK rSDS-PrP^Sc^ oligomers, as observed previously when prion-infected cells were treated with A6. Brain tissue samples, with normalized protein amounts, were PK-digested during 1 h at 37 °C and reactions were stopped by addition of a protease inhibitor cocktail. Samples were analysed by immunoblotting, using the SAFmix anti-PrP antibodies, as we showed that this mix specifically recognized the rSDS-PrP^Sc^ oligomers^15,26^. Brain homogenates of animals treated with A6 at 5 mg/kg did not revealed the presence of PK rSDS-PrP^Sc^ oligomers (Fig. 4a), and there is no difference in signal intensity between A6-treated animals and control samples either from non-treated (CTR), or DMSO-treated animals. Thus, A6 compound at a concentration of 5 mg/kg is not able to induce detectable PK rSDS-PrP^Sc^ oligomers *in vivo* suggesting that the shorter survival time observed in this group is not linked to these oligomeric species. No difference in the monomeric bands of PrP^Sc^ (20–37 kDa) is seen as all the brain homogenates analysed are at the terminal stage of the disease, which is consistent with the PET-blots at the terminal stage of the disease (Fig. 2c,3–5).

### PK-resistant PrP^Sc^ oligomers are clearly detectable in the brain of mice treated with 20 mg/kg of A6

Brain homogenates of animals treated with A6 at 10 and 20 mg/kg were also analysed for the presence of PK rSDS-PrP^Sc^ oligomers (Fig. 4b). For each sample, protein levels were measured and normalized in order to have equivalent protein amounts. Immunoblots were performed on samples, after PK digestion as described above. Immunoblots showed that brains from animals treated with higher concentrations of A6 present detectable levels of PK rSDS-PrP^Sc^ oligomers. Interestingly, the levels of PK rSDS-PrP^Sc^ oligomers are proportional to the concentration of A6: much more oligomers are present in the animals treated with 20 mg/kg of A6 compared to those treated with 10 mg/kg. Regarding the control groups treated with DMSO, traces of PK rSDS-PrP^Sc^ oligomers were observed corresponding to background levels (Fig. 4b). The loading charge control was done using an anti-β-actin antibody and confirmed that all samples were equivalently loaded on the gel (Fig. 4b). Animals non-inoculated with prions that were either treated with DMSO, or with 20 mg/kg of A6, did not present any PrP^Sc^ as expected. No difference in the monomeric bands of PrP^Sc^ (20–37 kDa) is seen as all the brain homogenates analysed are at the terminal stage of the disease, excepted for the asymptomatic mouse treated with 10 mg/kg that presented less monomeric PrP^Sc^ as expected (Supplementary Fig. S4). In order to confirm the presence of PK rSDS-PrP^Sc^ oligomers in A6-treated mice at 20 mg/kg, we analysed brain homogenates of five different animals sacrificed at the terminal stage of the disease (Fig. 5). The immunoblot showed that all animals analysed exhibit these PK rSDS-PrP^Sc^ oligomers illustrating the fact that treatment with higher doses of A6 can induce PK rSDS-PrP^Sc^ oligomers *in vivo*.

### Above a threshold of 1 mM, A6 induces a strong precipitation of PrP^Sc^ with appearance of rSDS-PrP^Sc^ oligomers in all fractions

The *in vivo* effects observed are visibly dependent on the concentration of A6 used. A6 compound is structurally related to another one, named MR100 that possesses a strong capacity to precipitate and aggregate prions *in vitro* with formation of rSDS-PrP^Sc^ oligomers on western blot. These properties have led to a diagnosis assay, called RCA (Rapid centrifugation assay) avoiding PK digestion step^26^. Due to the structural analogy between MR100 and A6 (Fig. 6a), and because A6 is able, as MR100, to induce rSDS-PrP^Sc^ oligomers on western blot, we believed that A6 could have the capacity to precipitate and aggregate prions *in vitro*. Thus, we have tested on a prion-infected brain homogenate, a range of concentrations of A6 (from 0.25 mM to 1.5 mM) using the same RCA protocol we previously developed with MR100, and by replacing MR100 compound by A6^26^ (Fig. 6). After 1 hour of incubation of the brain homogenate with the molecule, samples were centrifuged briefly at 8 000 g for 5 min, and pellets (P) and supernatants (S) were analysed on western blot. We used MR100 as a positive control of the aggregation, and the orange precipitate is clearly visible in the tube after centrifugation (Fig. 6b). A yellow precipitate is also clearly distinguished in A6 treated samples, which size is proportional to the concentration used (Fig. 6b), suggesting that A6 has the capacity to aggregate prions such as MR100. Western blot showed that consecutive to MR100 incubation, prions are mainly concentrated in the pellet fraction, with formation of rSDS-PrP^Sc^ oligomers (Fig. 6c). DMSO, used with an equivalent volume of the highest concentration of A6 (150 μL), was used as a negative control. Results showed that the solvent is not able to precipitate prions in the pellet and to form rSDS-PrP^Sc^ oligomers. Western blot analysis showed that at low concentrations (0.25 to 0.5 mM), prions mostly remained in the supernatant as soluble species with the presence of soluble rSDS-PrP^Sc^ dimers (Fig. 6c, left white square). At 1 mM of A6, an aggregation threshold occurred and immunoblot revealed the presence of rSDS-PrP^Sc^ oligomers in both supernatant and pellet (Fig. 6c). For concentrations above 1 mM, rSDS-PrP^Sc^ oligomers are more abundant in the pellet (Fig. 6c, right white square). Thus, A6 exhibit aggregation capacity towards prions, which is concentration-dependent. In order to estimate the proportion of rSDS-PrP^Sc^ oligomers within and between doses, we performed densitometry analyses on several independent RCA gels. Then, the ratio of rSDS-PrP^Sc^ oligomers in the pellet versus oligomers in the supernatant was calculated for each concentration and presented as a histogram (Fig. 6d). Results showed that the proportion of oligomers in the pellet versus oligomers in the supernatant is about 10 times higher at high doses (ratio of 1.7) compared to low doses (ratio of 0.16) (one-way ANOVA, **p-value = 0.0086).

This aggregation property of A6 compound likely reflects the difference observed *in vivo* regarding the incubation time and the rate of accumulation of prions in the brain of animals. Interestingly, RCA aggregation experiment with normal brain homogenate as a control was performed to see if A6 could also precipitate the normal PrP^C^ (Supplementary Fig. S4), as previously described for MR100 compound^26^. Pictures of the tubes showed that A6 pellets are visible but much smaller compared to the assay with infected brain homogenates (Fig. 6b), and the size of the pellets is not concentration-dependent (Supplementary Fig. S5). Remarkably, the immunoblot analysis showed that A6 is not able to precipitate PrP^C^ (Supplementary Fig. S5), by contrast to MR100 compound as described previously^26^.

## Discussion

Public concerns over the use of synthetic pesticides are growing since many studies have now shown their impact on human health including different kinds of cancers and neurodegenerative disorders^1,32–34^. Over the last decade, a new environmental movement in the occidental countries, promoting an organic agriculture has allowed the use of some botanical pest control and favoured the rebirth of botanical biopesticides^19^. Among them, the α-terthienyls (α-Ter), also called thiophenes, found in abundance in the roots of *Tagetes* species (family of Asteracea), are a powerful class of insecticides very efficient against larvae of several species of mosquitos^18–20^. Because regulations regarding the natural insecticides appeared softer than for the synthetics, one can also ask whether biopesticides are harmless or not for the human health.

**Figure 7:**
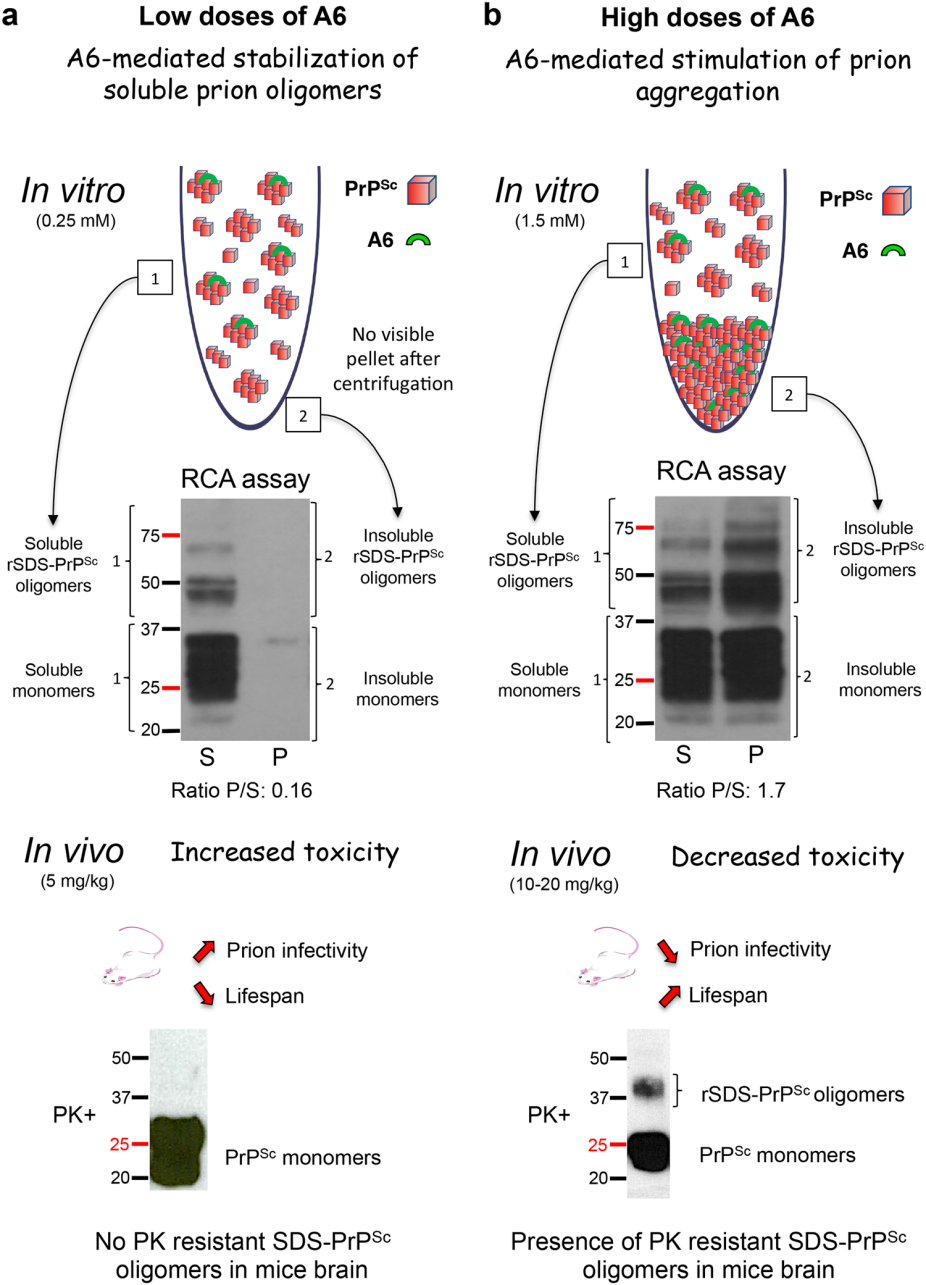
Schema illustrating the dual effect of A6 biopesticide on prion aggregation and propagation. (**a**) A6-mediated stabilization of soluble prion oligomers at low doses. Incubation of prion-infected brain homogenate with a low dose (0.25 mM) of A6 *in vitro*, allows formation of soluble rSDS-PrP^Sc^ oligomers (50–75 kDa) (1) that remains in the supernatant (S), whereas insoluble rSDS-PrP^Sc^ oligomers concentrated in the pellet fraction (P) are not formed (2). *In vivo* treatment of mice with a low dose of A6 (5 mg/kg) decreases animals’ survival time and increases prion amyloid deposits suggesting that soluble rSDS-PrP^Sc^ oligomers are toxic. These species are not visible in 5 mg/kg A6-treated mice brains. (**b**) A6-mediated stimulation of prion aggregation at high doses. Incubation of prion-infected brain homogenate with the highest dose (1.5 mM) of A6 *in vitro*, allows formation of insoluble rSDS-PrP^Sc^ oligomers (50–75 kDa) (2) mainly in the pellet fraction (P). *In vivo* treatment of mice with higher doses of A6 (10 and 20 mg/kg) increases animals’ survival time and decreases prion amyloid deposits suggesting that insoluble rSDS-PrP^Sc^ oligomers are less toxic. These species are quite abundant in mice brains treated with 20 mg/kg of A6, suggesting that insoluble rSDS-PrP^Sc^ oligomers have a rather protective role.

In the present study, we showed a dual effect of A6 (Fig. 7), an analogue of α-Ter described for its herbicidal property, on prion propagation depending on the doses administered.

At the lowest dose of 5 mg/kg: the treatment with A6 compound accelerates the prion pathology, with appearance of earlier PrP^Sc^ deposits and the survival time of mice is significantly reduced. However no PK rSDS-PrP^Sc^ dimers could be detected in brains (Fig. 7a)^15^. To analyse the aggregation property of A6 compound on prions, we performed an *in vitro* assay, the RCA described previously^26^. Our results on RCA showed that at the lowest dose of 0.25 mM, A6 can induce rSDS-PrP^Sc^ oligomers that were detected in the supernatant but not in the pellet, which means that those species are soluble (Fig. 7a). Thus, at lower concentrations, A6 rather promotes soluble rSDS-PrP^Sc^ oligomers (ratio pellet/supernatant: 0.16, Fig. 6d), usually considered as neurotoxic species, which is consistent with our *in vivo* results of A6-treated mice at the lowest dose of 5 mg/kg exhibiting shorter incubation time.

At the highest doses of 10 and 20 mg/kg, we observed an opposite effect: A6 treatment delays the appearance of PrP^Sc^ deposits in mice brains and their survival times are significantly increased (Fig. 7b). We could also detect PK rSDS-PrP^Sc^ oligomers in all brain homogenates of mice treated with 10 or 20 mg/kg that were analysed. The detection of PK rSDS-PrP^Sc^ dimers in the group of mice treated with 10 and 20 mg/kg of A6, but not in the group treated with 5 mg/kg, strongly suggests that these oligomeric species are implicated in the increasing survival time of animals. Those PK rSDS-PrP^Sc^ oligomers could be either amorphous aggregates trapping a part of prion infectiosity or non-toxic aggregates not able to propagate prions. Our *in vitro* results obtained by the RCA method^26^, are in agreement with this hypothesis since at high doses of A6, above 1 mM, prions aggregate into a yellow precipitate, and high levels of insoluble rSDS-PrP^Sc^ oligomers were detected in the pellet (Ratio Pellet/Supernatant: 1.7, Fig. 6b–d), which is consistent with our *in vivo* results. Although the range of concentrations used *in vivo* and *in vitro* are quite different because the systems used cannot be comparable, one can notice that a ratio of 4 is observed. Indeed, we observed opposite effects *in vivo* between 5 and 20 mg/kg on prion propagation, while the same ratio is observed *in vitro* between 0.25 mM and 1 mM switching from soluble to insoluble PrP^Sc^ oligomers.

In addition, we also detected such kind of insoluble PK rSDS-PrP^Sc^ oligomers in different prion strains, including sporadic (sCJD) and new variant CJD^26^ by using MR100 compound, structurally related to A6, with four thiophene cycles instead of two. Remarkably, we showed that levels of rSDS-PrP^Sc^ oligomers are correlated with the length of the symptomatic phase of sCJD patients^26^. The more they are abundant in the brain tissue, the longer is the symptomatic phase of the disease, as if they have a protective role, which is in agreement with our present data of A6-treated mice at high doses. Interestingly, Herrmann *et al*.^35^ have shown that Luminescent Conjugated Oligothiophene (LCOs), molecules containing several thiophenes (n = 4–7) have the ability to generate SDS-stable PrP^Sc^ oligomers, very similar to A6, in a transgenic mouse model of prions. Administration of polythiophenes to the brain of prion-infected mice via osmotic minipump led to a survival of 80% and showed an anti-prion activity against mice and hamster strains^35^.

Regarding the mechanism of action, we used the fluorescence property of A6 to study the interaction of the compound with either soluble or fibrillar recPrP. A6 is a hydrophobic compound and in aqueous environment its fluorescence is quenched. Upon binding with PrP fibrils, either mouse or Hamster R-fibrils, the environment of A6 is less aqueous and the intensity of the signal is doubled illustrating the interaction protein-molecule. Epifluorescence studies also showed that R-fibrils could be labelled in blue by A6, in a similar way as ThT labelled R-fibrils in green (data not shown). ThT is a referenced fluorescent dye known to interact with beta-sheets of amyloid fibrils suggesting that A6 has a potential to be a tracer to study PrP fibrils. Interestingly, A6 is an analogue of LCOs with two thienyl cycles, and previous studies on LCOs showed that the fluorescent properties of these molecules are exacerbated upon binding with amyloid fibrils, not only PrP but also beta-amyloid peptide, and they have a strong potential as tracer of amyloid deposits in brain tissue^36^. It could be interesting in the future to see if A6 can label other amyloid fibrils such as beta-amyloid peptide and eventually explore its potential as a tracer of amyloid fibrils in Alzheimer’s disease tissues.

Our results showed that A6 interacts with PrP fibrils, but how to explain the precipitation observed and the formation of rSDS-PrP^Sc^ oligomers both *in vitro* and *in vivo*?

It was previously described that α-Ter generates oxygen radical species (superoxide anion radical, singlet oxygen) and α-Ter radical^18^. Because A6 interacts with PrP, we can make the hypothesis that A6-PrP• radicals can be generated and re-association of A6-PrP• radicals can lead to rSDS-PrP^Sc^ oligomers covalently bound. Preliminary mass spectrometry experiments with mouse recombinant PrP oligomers did not allow us to demonstrate it (data not shown, Human Rezaï, personal communication). However many reasons such as the fact that the fibrils tested may not be adapted or the necessity of other cofactors could explain it. It has been demonstrated that α-Ter also has the capacity to inhibit several enzymes such as superoxide dismutase and acetylcholinesterase (AChE) both *in vitro* and *in vivo* leading to important consequences in nervous, respiratory and digestive systems of mosquito larvae^18^. Remarkably, a recent study conducted by Torrent *et al*.^37^ showed that: (*i*) AChE interacts with PrP monomers and aggregates them; (*ii*) upon AChE interaction with PrP fibrils, a complete rearrangement of the fibrils occurred and the enzyme looses its activity which worsen the cytotoxicity of prions; and (*iii*) in heterozygous AChE knock-out mice (TgAChE^+/−^) presenting 25% decrease in the levels of AChE enzyme, a prolonged survival time was observed when these mice were challenged by prions compared to control wild-type (WT) animals^37^. Thus, we cannot exclude that A6 can impair the homeostasis of AChE, as well as a possible competition of A6 in the complex formed between AChE/PrP. It is likely that a combination of several mechanisms explains the observed phenotype in the mice treated with low or high doses of A6 compound and further experiments will be necessary in the future to clarify them. Our results showed that the α-Ter analogue A6 could trigger soluble or insoluble rSDS-PrP^Sc^ oligomers that worsen or delay the prion pathology, depending on the doses used. The fact that the lower doses are more toxic than the high ones, should alert us regarding the pesticides (synthetics or botanical pesticides). Since many pharmacological molecules are originally plant-derived extracts, we should revise our toxicological assays towards low doses for the future development of therapeutics in neurodegenerative disorders.

## Materials and Methods

### Ethics statement and animal housing

This project follows the specific French national guidelines on animal experimentation and animal well-being and was approved by the French National Ethic Committee for Animal Experimentation (Nr. CE-LR-11001). WT mice (4–6 weeks old) were housed in an A3/L3 biosafety facility, in an enriched environment with cotton pads placed in their cages. Animals had free access to water and food, and were fed under a standard chow diet (A03) (SAFE Diets, France).

### Biological reagents and antibodies

Pefabloc and proteinase K were purchased from Roche Diagnostics (Mannheim, Germany). The protein assay kit based on the bicinchoninic acid (BCA) was purchased from Pierce (Thermofisher Scientific, Saint Herblain, France). For immunoblotting analyses, we used a mix (SAF mix) of three anti-PrP antibodies (SAF60, SAF69 both recognize epitope 157–161 of HuPrP and SAF70 recognizes epitope 156–162 of HuPrP) that were kindly provided by Dr. Jacques Grassi (CEA, Saclay, France). The SAF69 antibody is critical for detection of PrP oligomers. The SAF84 antibody (epitope 161–170 of HuPrP) used for PET-blot analyses was purchased from SpiBio (Montigny-le-Bretonneux, France). Secondary antibodies were from Jackson ImmunoResearch (West Grove, PA, USA). All other chemicals and antibodies were purchased from Sigma (Paris, France).

### Chemical reagents

The thienyl pyrimidine compounds A6, α-Ter, P30, A12, A14, A18 and A51 that exhibit or not rSDS-PrP^Sc^ oligomer-inducing activity^15,26^ were purchased from Maybridge (Cornwall, United Kingdom) and Key Organics Limited (Cornwall, United Kingdom). Stock solutions were prepared at 5 mM and drugs were solubilized in DMSO according to the suppliers’ recommendations. A6 was heated at 80 °C for 10–20 min to reach complete solubility. For the synthesis of MR100 (6,6′-(2,2′:5′,2″:5″,2″′-quaterthiophene-5,5″′-diyl)bis(1,3,5-triazine-2,4diamine)), reactions were carried out as described previously^26^. Drugs are stored in the dark at room temperature.

### Cell culture and cell screening assay

The mouse neuroblastoma cell line N2a was purchased from the American Type Culture Collection (ATCC CCL131). The N2a58 subclone, which over-expresses mouse PrP (MoPrP), was chronically infected with the mouse-adapted scrapie strain 22L (N2a58/22L cells), as described by Nishida *et al*.^38^. N2a58/22L cells were cultured as described previously^15,26^. For drug screening, N2a58/22L cells (~10^6^ cells/25-cm^2^ flasks) were incubated with the compounds (A6, P30, A12, A14 and A18) at a final concentration of 20 μM (corresponding to 20 μL of drug at 5 mM or 20 μL of solvent alone DMSO, in 5 mL of medium for a T25 plate) for 4 days. At confluence, cells were lysed in 400 μL lysis buffer (0.5% NP-40, 0.5% Deoxycholate, 10 mM Tris-HCl pH 8, 100 mM NaCl).

For a fast screening of drugs, 500 μL of N2a58/22L cell lysates were incubated with 50 μL of drug (P30, A6, MR100, α-Ter, and A21) at a final concentration of 0.5 mM, for 1 hour at room temperature and processed as described below for western blotting analysis.

### Immunoblotting

Protein concentration in cellular lysates was measured using the BCA assay. For western blots, all samples were normalized regarding their protein amounts and volumes. Normalized samples were digested with 20 µg/mL of proteinase K (PK) at a ratio of 1:25 (protease to protein) at 37 °C for 1 h. Digestion was stopped with 1X Complete (Roche, Boulogne-Billancourt, France) and samples were centrifuged at 20 000 g at 4 °C for 30 min. Pellets were dissolved in 20 µL lysis buffer and 20 µL 2X loading buffer (0.1 M DTT, 3% SDS, 20% glycerol, 0.4 M Tris-HCl pH 7.4 and bromophenol blue), then boiled for 3 min before loading on 12% SDS-PAGE Criterion precast gels (Biorad, Marne-La-Coquette, France). Western blotting was performed according to standard procedures and MoPrP was detected with the SAF mix, as mentioned above (Cf. § “Biological reagent and antibodies”). Membranes were revealed with an ECL solution (Luminata Crescendo western HRP substrate, Millipore, Guyancourt, France).

### Purification of prion protein and formation of amyloid fibrils

Full-length recombinant mouse PrP encompassing residues 23–230 (MoPrP23–230) was expressed in *E. coli* and purified as described previously^39^. The purified recombinant MoPrP23-230 mostly folded into α-helices was confirmed by SDS-PAGE and electrospray mass spectrometry to be a single species with an intact disulfide bond and correct molecular weight as previously described by Ayrolles-Torro *et al*.^15^. After purification, MoPrP was stored in lyophilized form at −20 °C. Amyloid fibrils using full-length MoPrP23-230 were formed using the manual setup protocol of Breydo *et al*.^40^, and fibrils formation was monitored after collecting aliquots to which 10 μM of thioflavin T (ThT) were added^41^. ThT fluorescence emission spectra were recorded after excitation at 450 nm as previously described^25,40^. The quality of freshly made fibrils was also confirmed by transmission electron microscopy as described previously^24^. Fibrils were stored at 4 °C until use for further analyses. Two amyloid strains: hamster S-fibrils (for “shaking”) and hamster R-fibrils (for “rotation”) were kindly provided by Dr. Ilia Baskakov (University of Maryland School of Medicine, Baltimore, MD, USA). These hamster fibrils were produced *in vitro* using full-length hamster PrP, according to the manual format protocol as described by Makarava *et al*.^29^.

### Fluorescence spectra

The fluorescent properties of A6 compound were described previously (Nasri *et al*. 2016). The A6 molecule has a λ_ex_ = 372 nm and a maximal peak of emission of fluorescence at 460 nm. Interaction studies between PrPs (either α-folded or fibrils) and A6 were done by fluorescence and all spectra were recorded from 200–600 nm using a fluorimeter FluoroMax2 (JobinYvon Spex, Tokyo, Japan). Lyophilised MoPrP23-230 was solubilized at 0.5 mg/mL (0.2 mM) in 50 mM MES buffer pH 6, and filtrated on 0.2 μm filter. The α-folded MoPrP23-230 stock solution freshly made and the MoPrP fibrils stored at 4 °C were diluted in 50 mM MES buffer pH 6, at a final protein concentration of 4.4 μM, and mixed with 50 μM of A6 compound, 1% DMSO, during 2 h at 25 °C. Hamster S- or R-fibrils (4.4 μM) were incubated with 40 μM of A6 compound, diluted in 50 mM MES buffer pH 6, 1% DMSO during 2 h at room temperature. Then, fluorescence experiments were conducted as described previously by excitation of the A6 compound at 372 nm.

### Aggregation assay

Brains from a terminally sick mouse (infected with 22L prions) or from a non-infected mouse were homogenized in 10% (w/v) sterile PBS solution using microbead-containing tubes and a Ribolysor apparatus (Biorad, Marnes la Coquette, France). Tubes were shaken for 45 s and homogenates were collected with an insulin syringe to obtain a homogeneous suspension, and immediately frozen at −80 °C. Then, we performed the aggregation test with a range of concentrations of A6 by following the protocol of the rapid centrifugation assay (RCA) initially described with the MR100 compound by Imberdis *et al*.^26^. Briefly, 50 µL of 22L-infected brain extracts (10% w/v) were diluted in PBS - 2% Sarkosyl and incubated with 1.5 mM (150 μL) of MR100, as a positive control of RCA, or a range of concentrations of A6 from 0.25 mM (25 μL) to 1.5 mM (150 μL), in a final volume of 0.5 mL for 1 h at room temperature. A sample incubated with 150 μL of DMSO was used as a negative control of RCA, corresponding to the highest concentration used (1.5 mM). Samples were then centrifuged in a benchtop centrifuge (Eppendorf) at 8 000 g for 5 min. Supernatants were removed and 50 μL aliquots were mixed with an equal volume of 2X loading buffer. Pellets were suspended in 50 μL of PBS -2% Sarkosyl and mixed with an equal volume of 2X loading buffer. Supernatants and pellets were analysed by western blotting using SAF mix antibodies.

### Prion inoculations

Groups of C57Bl/6J females (n = 10–15 animals/group) were inoculated with 5 μL of 1% brain homogenate infected with 22L prion strain into their striatum using a stereotaxic frame (Kopf Instruments, Tujunga, CA, USA) with the following coordinates: L: 2.0 mm; A/P: 0 mm; and D/V: −3.0 mm^42^. For control female mice (n = 10) non-inoculated with prions, 5 μL of PBS was injected into the striatum using the same method and coordinates. In a first series of experiments, mice were treated by intra-peritoneal route with 5 mg/kg of A6 compound (50 μL at 3 mg/mL of A6) or with 50 μL of pure DMSO alone, for 5 weeks starting one week after prion infection. In a second series of experiments, mice were treated with higher concentrations of A6 compound by intra-peritoneal route: either at 10 mg/kg (100 μL at 3 mg/mL A6) or 20 mg/kg (100 μL at 6 mg/mL A6) or with 100 μL of pure DMSO alone, for 12 weeks starting one week after prion infection. For the highest treatment of A6 (10 or 20 mg/kg), the volume injected to mice was doubled due to the poor solubility of A6 (especially at 6 mg/mL). During the experiments, groups of five mice were housed in cages placed in a ventilated protective room. Mice were scored positive for prion disease when three signs of neurologic dysfunction were observed and when progressive deterioration (according to 16 diagnostic criteria) was apparent, as described previously^43,44^. Once clinical signs were detected, the animals were observed daily and killed *in extremis*, as described previously^43,44^. Their brains were removed and immediately frozen at −80 °C for homogenization, or fixed in AntigenFix (Diapath, France) for immunohistochemical analysis.

### Immunohistochemistry

Brain tissues were fixed in AntigenFix solution (Diapath, France) for 24 h. Then, they were decontaminated for 30 min in formic acid solution according to the protocol described by Andréoletti *et al*.^45^ and stored in 100 mM phosphate buffer at pH 7.4 with 0.02% sodium azide. Samples were dehydrated in graded ethanol, cleared in cedar oil and embedded in paraffin. Using a microtome, 6 μm frontal sections were cut and mounted on Superfrost Plus slides (Microm France, Francheville). Sections were dewaxed and stained with hematoxylin and eosin (HE) as described previously^46^. Immunolabelling with anti-GFAP (1:500; Dako, Les Ulis, France) antibodies was performed according to the instructions provided with the Strept ABC Complex Kit. Labelling was visualized using 3–3′-diaminobenzidine chromogen solution (Sigma, France). For paraffin-embedded tissue blots (PET-blots), 6 μm frontal sections were cut using a microtome and placed on nitrocellulose membrane. After drying at 50 °C for 48 h, sections were dewaxed, digested with 25 µg/mL PK at 56 °C overnight and then denatured with 3 M guanidine thiocyanate for 10 min. Membranes were blocked with casein for 30 min. The SAF84 antibody was used to label PrP^Sc^ and the Vectastain ABC-AmP kit (Vector laboratories, USA) to reveal antibody binding.

### Softwares and statistical analyses

Kaplan-Meier survival curves were done using the GraphPad Prism software (La Jolla, CA, USA). The difference between curves was tested using the non-parametric Mantel-Cox test and Wilcoxon test, with a probability of 0.05 defined as a significant difference. Survival times are expressed as median values. Oligomer fractions were quantified using Fiji software (2.0 version 2.0; National Institutes of Health, Bethesda, MD). Statistical analyses were performed on GraphPad Prism software using a one-way ANOVA coupled with a Tukey’s multiple comparison test, with a probability of 0.05 defined as a significant difference.

### Equipment and settings

The blots presented in Fig. 1, 4–5 and in Supplementary Fig. S4 were revealed with Amersham Hyperfilm ECL (Thermo Scientific, Illkirch, France) by using a Konica medical film processor SRX-101 (Konica Minolta, Amsterdam, Netherlands).

### Data availability statement

The datasets generated during and/or analysed during the current study are available from the corresponding author on reasonable request.

## Electronic supplementary material

Supplementary Figures S1 to S7

Original blots

## References

[1] Chin-Chan M, Navarro-Yepes J, Quintanilla-Vega B (2015). Environmental pollutants as risk factors for neurodegenerative disorders: Alzheimer and Parkinson diseases. Front. Cell. Neurosci..

[2] Schwarzenbach RP (2006). The challenge of micropollutants in aquatic systems. Science.

[3] United Nations Educational, Scientific, and Cultural Organization, World Water Assesment programme. Water for people, Water for Life-the United Nations World water Development Report. Bergham Books, Barcelona (2003).

[4] Nougadère A (2012). Total diet study on pesticide residues in France: levels in food as consumed and chronic dietary risk to consumers. Environ. Int..

[5] Richardson JR (2014). Elevated serum pesticide levels and risk for Alzheimer disease. JAMA Neurol..

[6] European Decision n°2008/217/EC : Commission Decision of 10 April 2008 concerning the noninclusion of rotenone, extract from equisetum and chinin-hydrochlorid in Annex I to Council Directive 91/414/EEC and the withdrawal of authorisations for plant protection products containing these substances. OJ L 108 (2008).

[7] Judgment of the court of first instance. Directive 91/414/EEC – Plant protection products – Paraquat as an active substance – Marketing authorisation – Authorisation procedure – Protection of human and animal health. Case T-229/04. European Court Reports 2007 II-02437 (2007).

[8] Tanner CM (2011). Rotenone, paraquat, and Parkinson’s disease. Environ. Health Perspect..

[9] van der Mark M (2012). Is pesticide use related to Parkinson disease? Some clues to heterogeneity in study results. Environ. Health Perspect..

[10] Betarbet R (2000). Chronic systemic pesticide exposure reproduces features of Parkinson’s disease. Nat. Neurosci..

[11] Gordon I, Abdulla EM, Campbell IC, Whatley SA (1998). Phosmet induces up-regulation of surface levels of the cellular prion protein. Neuroreport.

[12] Purdey M (1996). The UK epidemic of BSE: slow virus or chronic pesticide-initiated modification of the prion protein? Part 2: An epidemiological perspective. Med. Hypotheses.

[13] Brundin P, Melki R, Kopito R (2010). Prion-like transmission of protein aggregates in neurodegenerative diseases. Nat. Rev. Mol. Cell Biol..

[14] Acquatella-Tran Van BI, Imberdis T, Perrier V (2013). From prion diseases to prion-like propagation mechanisms of neurodegenerative diseases. Int. J. Cell Biol..

[15] Ayrolles-Torro A (2011). Oligomeric-induced activity by thienyl pyrimidine compounds traps prion infectivity. J. Neurosci. Off. J. Soc. Neurosci..

[16] Imberdis T, Ayrolles-Torro A, Verdier J-M, Perrier V (2013). Thienyl pyrimidine derivatives with PrP(Sc) oligomer-inducing activity are a promising tool to study prions. Curr. Top. Med. Chem..

[17] Friedman DCS, Friedman P (1995). A theoretical-study of 2,2″,5′,2″-Terthiophene (alpha-T) and its analogs.1. Correlation of electronic-structure and energies with herbicidal phototoxicity. J. Mol. Struct. Theochem.

[18] Nivsarkar M, Cherian B, Padh H (2001). Alpha-terthienyl: A plant-derived new generation insecticide. Curr. Sci..

[19] Arnason, J. T., Sims, S. & Scott, I. M. Natural products from plants as insecticides. Phytochem & Pharmacog, UNESCO ECLOSS Report (2012).

[20] Marles R (1995). Pharmacokinetics, metabolism and toxicity of the plant-derived photoxin alpha-terthienyl. Pharmacol. Toxicol..

[21] Loi n°2015-992 du 17 août 2015 relative à la transition énergétique pour la croissance verte, NOR:DEVX1413992L. *JORF* 14–263 (2015).

[22] Silveira JR (2005). The most infectious prion protein particles. Nature.

[23] Simoneau S (2007). *In vitro* and *in vivo* neurotoxicity of prion protein oligomers. PLoS Pathog..

[24] El Moustaine D, Perrier V, Smeller L, Lange R, Torrent J (2008). Full-length prion protein aggregates to amyloid fibrils and spherical particles by distinct pathways. FEBS J..

[25] El Moustaine D (2011). Amyloid features and neuronal toxicity of mature prion fibrils are highly sensitive to high pressure. J. Biol. Chem..

[26] Imberdis T (2016). A Fluorescent Oligothiophene-Bis-Triazine ligand interacts with PrP fibrils and detects SDS-resistant oligomers in human prion diseases. Mol. Neurodegener..

[27] Zechmeister L, Sease JW (1947). A blue-fluorescing compound, terthienyl, isolated from marigolds. J. Am. Chem. Soc..

[28] Nasri, A. *et al*. Neurotoxicity of a Biopesticide Analog on Zebrafish Larvae at Nanomolar Concentrations. *Int. J. Mol. Sci*. **17** (2016). 10.3390/ijms17122137 PMC5187937 27999363

[29] Makarava N, Baskakov IV (2008). The same primary structure of the prion protein yields two distinct self-propagating states. J. Biol. Chem..

[30] Ostapchenko VG (2010). Two amyloid States of the prion protein display significantly different folding patterns. J. Mol. Biol..

[31] Kim YS, Carp RI, Callahan SM, Wisniewski HM (1987). Incubation periods and survival times for mice injected stereotaxically with three scrapie strains in different brain regions. J. Gen. Virol..

[32] Gaspari L, Paris F, Jeandel C (2011). & Sultan, C. Peripheral precocious puberty in a 4-month-old girl: role of pesticides?. Gynecol. Endocrinol. Off. J. Int. Soc. Gynecol. Endocrinol..

[33] Gaspari L (2012). High prevalence of micropenis in 2710 male newborns from an intensive-use pesticide area of Northeastern Brazil. Int. J. Androl..

[34] Paris F, Gaspari L, Servant N, Philibert P, Sultan C (2013). Increased serum estrogenic bioactivity in girls with premature thelarche: a marker of environmental pollutant exposure?. Gynecol. Endocrinol. Off. J. Int. Soc. Gynecol. Endocrinol..

[35] Herrmann US (2015). Structure-based drug design identifies polythiophenes as antiprion compounds. Sci. Transl. Med..

[36] Klingstedt T (2013). The structural basis for optimal performance of oligothiophene-based fluorescent amyloid ligands: conformational flexibility is essential for spectral assignment of a diversity of protein aggregates. Chem. Weinh. Bergstr. Ger..

[37] Torrent J (2015). Interaction of prion protein with acetylcholinesterase: potential pathobiological implications in prion diseases. Acta Neuropathol. Commun..

[38] Nishida N (2000). Successful transmission of three mouse-adapted scrapie strains to murine neuroblastoma cell lines overexpressing wild-type mouse prion protein. J. Virol..

[39] Rezaei H (2000). High yield purification and physico-chemical properties of full-length recombinant allelic variants of sheep prion protein linked to scrapie susceptibility. Eur. J. Biochem..

[40] Breydo L, Makarava N, Baskakov IV (2008). Methods for conversion of prion protein into amyloid fibrils. *Methods Mol*. Biol. Clifton NJ.

[41] Naiki H, Higuchi K, Hosokawa M, Takeda T (1989). Fluorometric determination of amyloid fibrils *in vitro* using the fluorescent dye, thioflavin T1. Anal. Biochem..

[42] Paxinos, G. & Francklin, K. B. Paxinos and Franklin’s the Mouse Brain in Stereotaxic Coordinates. 4th ed. Elsevier: Academic Press (2012).

[43] Carlson GA (1986). Linkage of prion protein and scrapie incubation time genes. Cell.

[44] Scott M (1989). Transgenic mice expressing hamster prion protein produce species-specific scrapie infectivity and amyloid plaques. Cell.

[45] Andréoletti O. PrPSc Immunohistochemistry (ed. Lehmann, S., Grassi, J.). 82–96 (Tech. Prion, 2004). Available from: 10.1007/978-3-0348-7949-1_7.

[46] Toupet K (2008). Effective gene therapy in a mouse model of prion diseases. PloS One.

