# Supplementary material for: Low doses of bioherbicide favour prion aggregation and propagation in vivo
Source: Sci Rep. 2018 May 23;8:8023. doi: 10.1038/s41598-018-25966-9 (PMC5966510; doi:10.1038/s41598-018-25966-9)
Supplement: Supplementary file 1 — Supplementary Figures S1 to S7 [file 41598_2018_25966_MOESM1_ESM.pdf]

Title : Low doses of bioherbicide favour prion aggregation and propagation *in vivo*

Authors : Pierre-André Lafon, Thibaut Imberdis, Yunyun Wang, Joan Torrent, Mike Robitzer, Elisabeth Huetter, Maria-Teresa Alvarez-Martinez, Nathalie Chevallier, Laurent Givalois, Catherine Desrumaux , Jianfeng Liu and Véronique Perrier.

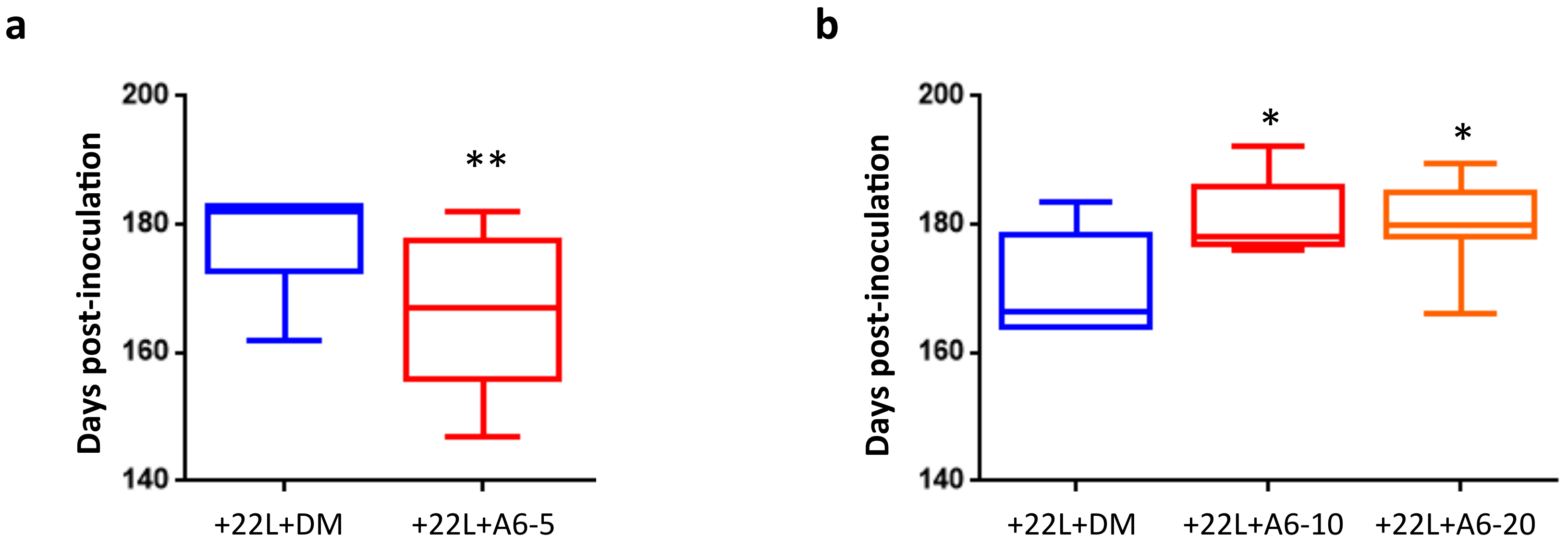

Supplementary Figure S1. Box-and-whiskers representation of the lifespan of animals treated with various concentrations of the bioherbicide A6

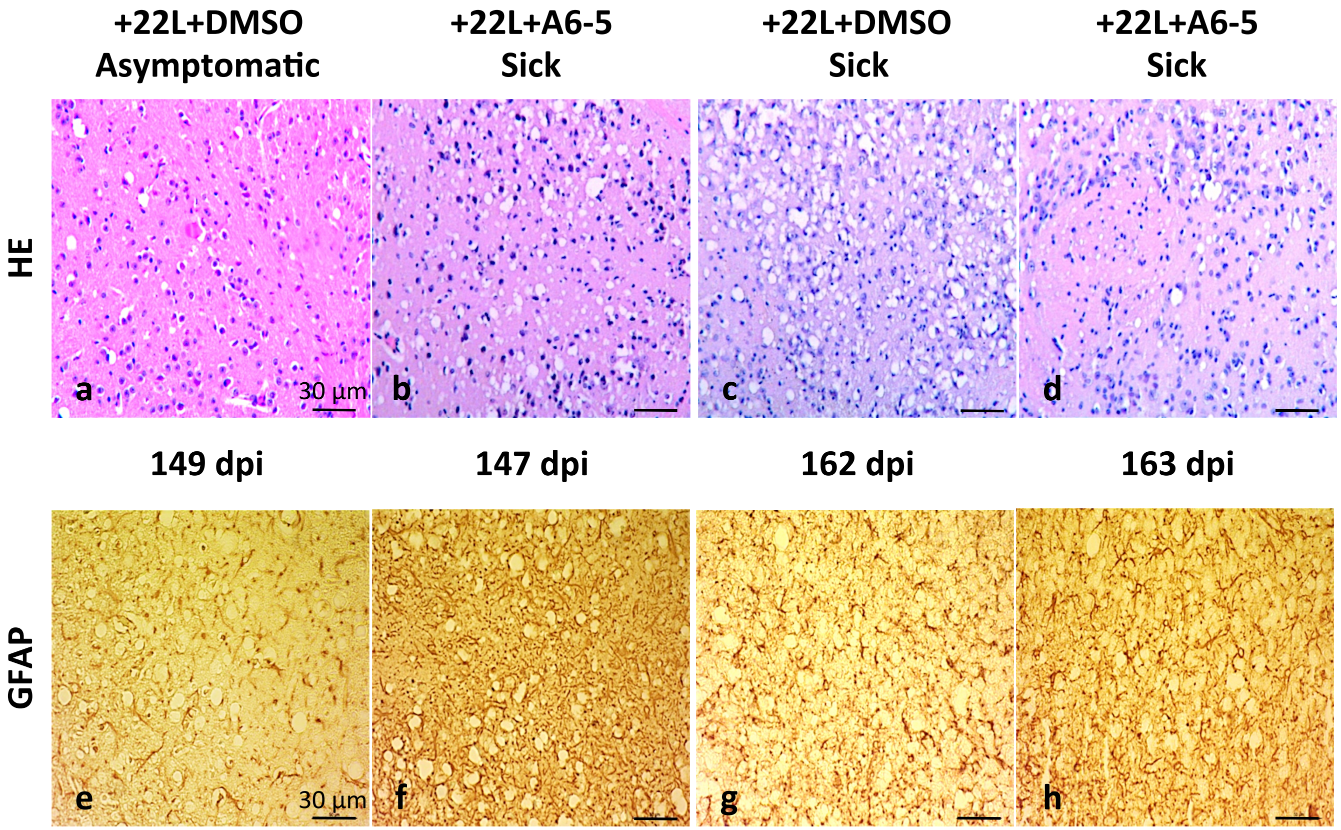

Supplementary Figure S2. Analyses of spongiosis and astrogliosis in brain tissue sections of mice treated with 5 mg/kg.

a

+22L+DMSO
Sick
164 dpi

+22L+A6-10
Asymptomatic
163 dpi

+22L+A6-20
Asymptomatic
162 dpi

HE

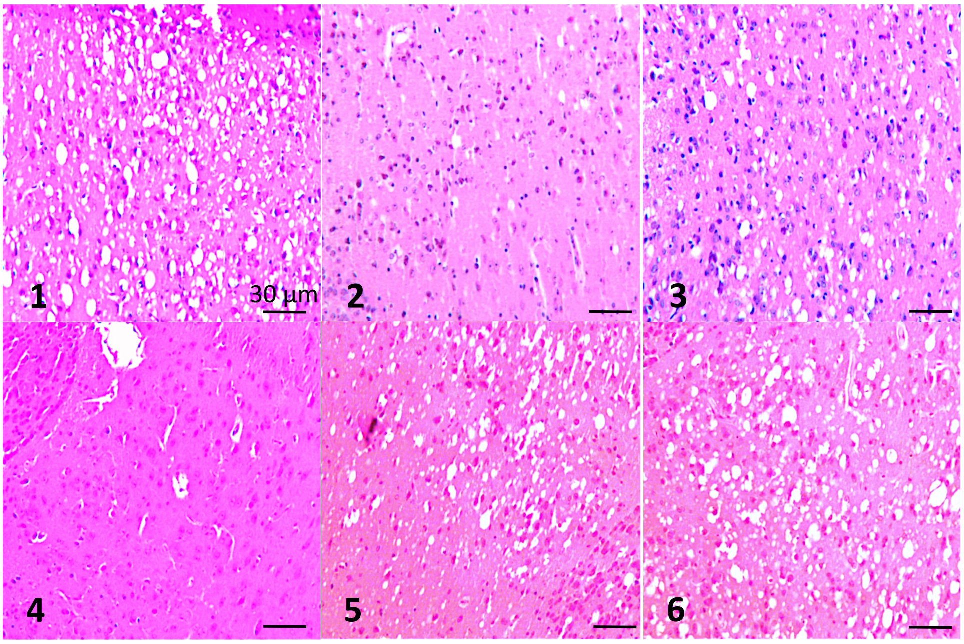

-22L+A6-20
Healthy
166 dpi

+22L+A6-10
Sick
186 dpi

+22L+A6-20
Sick
185 dpi

b

+22L+DMSO
Sick
164 dpi

+22L+A6-10
Asymptomatic
163 dpi

+22L+A6-20
Asymptomatic
162 dpi

GFAP

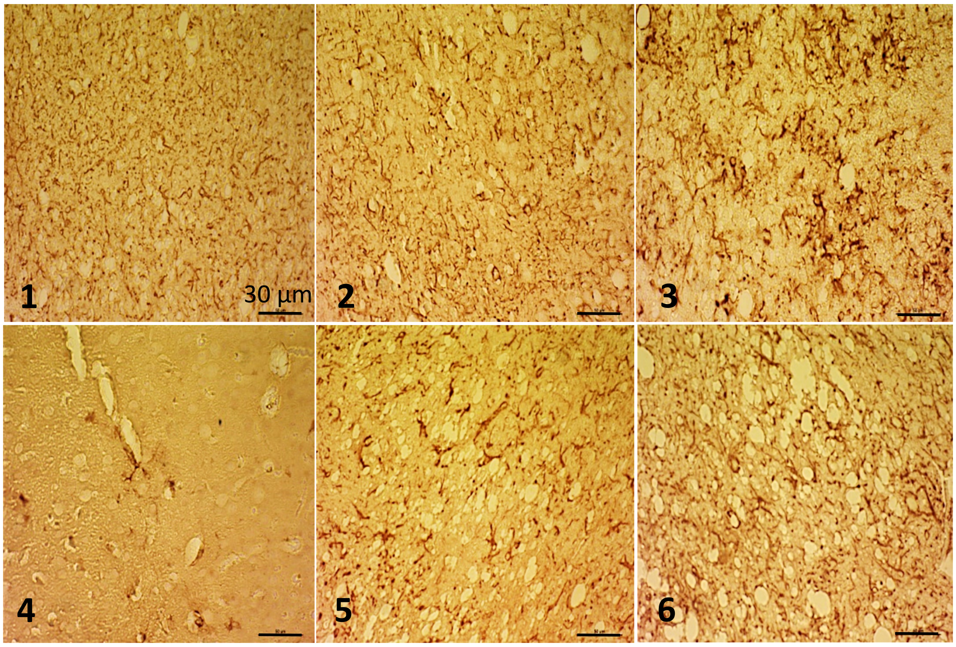

-22L+A6-20
Healthy
166 dpi

+22L+A6-10
Sick
186 dpi

+22L+A6-20
Sick
185 dpi

Supplementary Figure S3. Analyses of spongiosis and astrogliosis in brain tissue sections of mice treated with 10 and 20 mg/kg of A6.

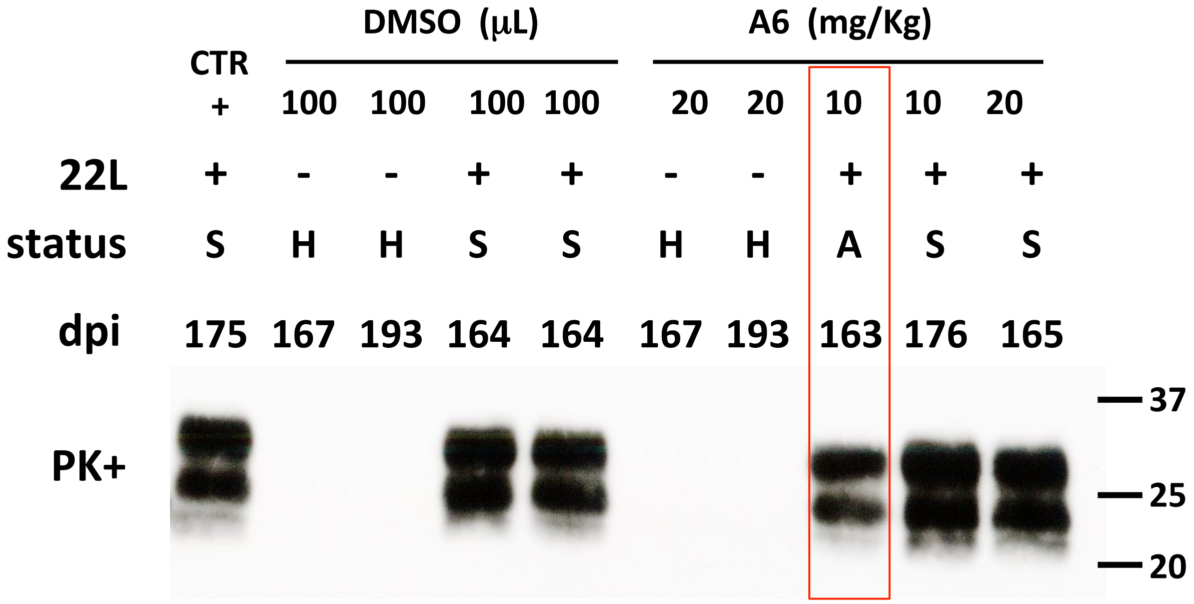

Supplementary Figure S4. Less exposed blot presented in Figure 4b.

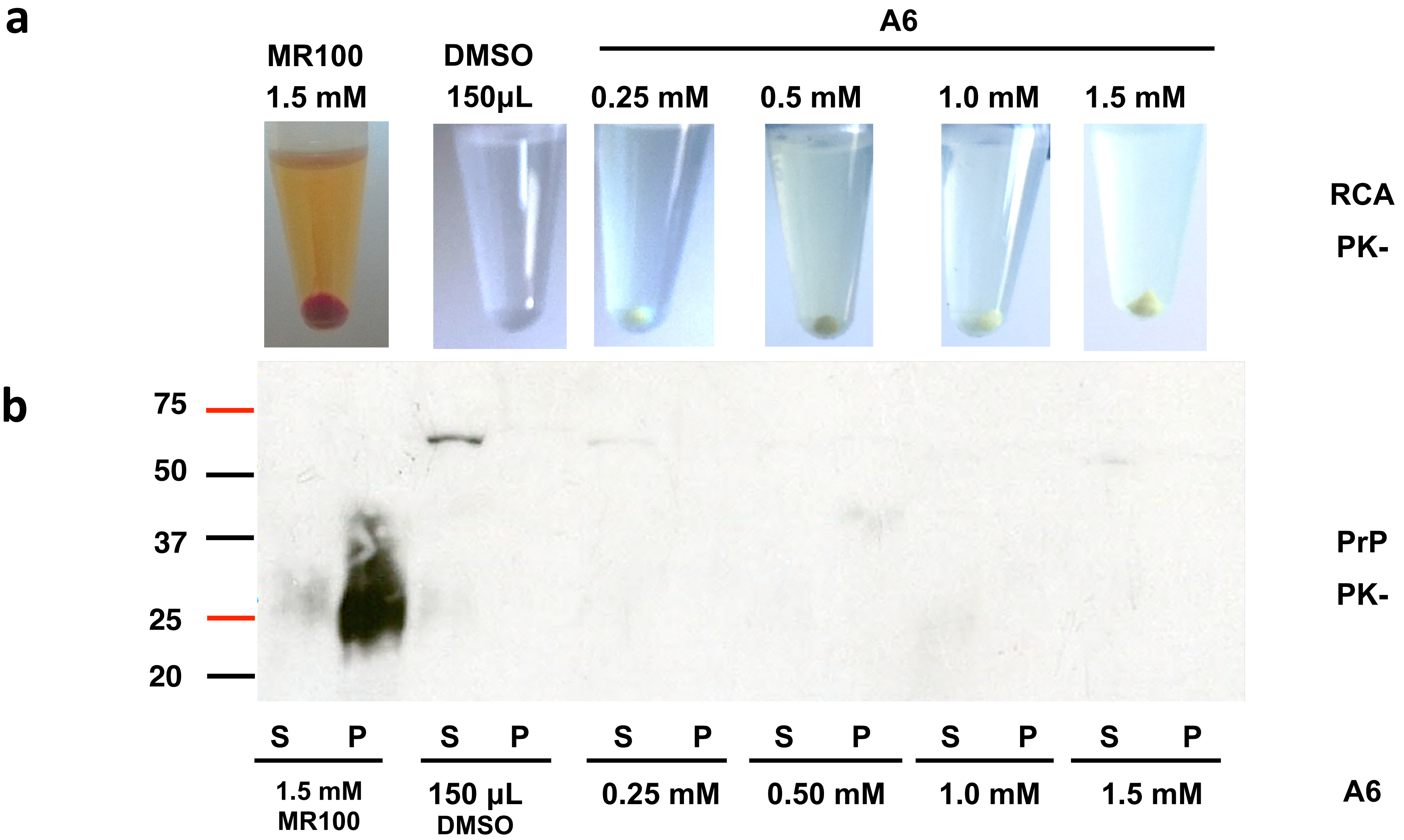

Supplementary Figure S5. A6 cannot precipitate PrP^C in non-infected brain homogenate.

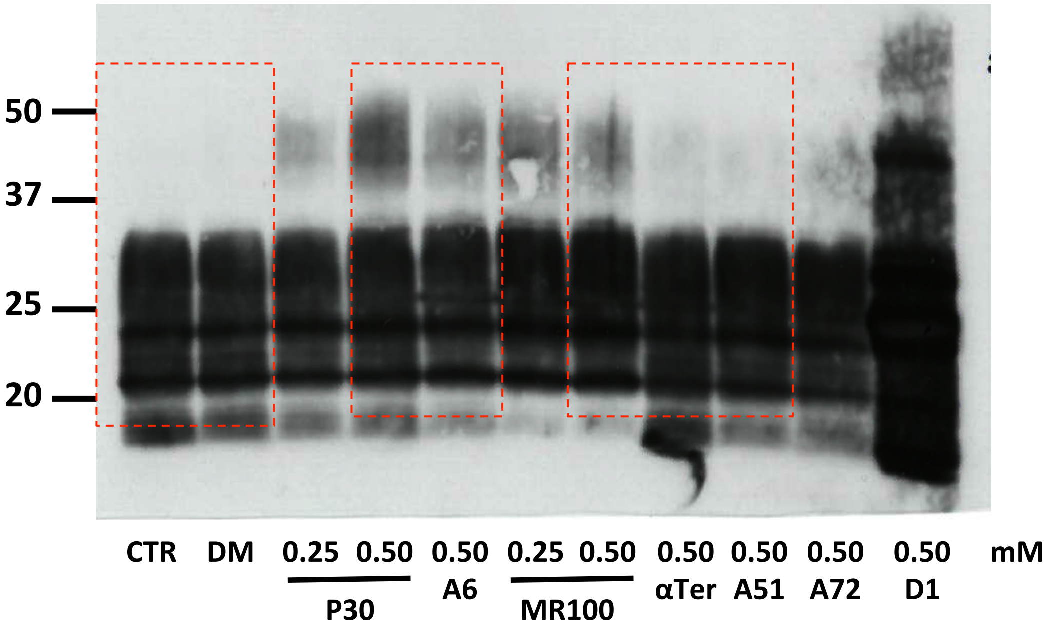

Supplementary Figure S6. Full-length blot showed in Figure 1a.

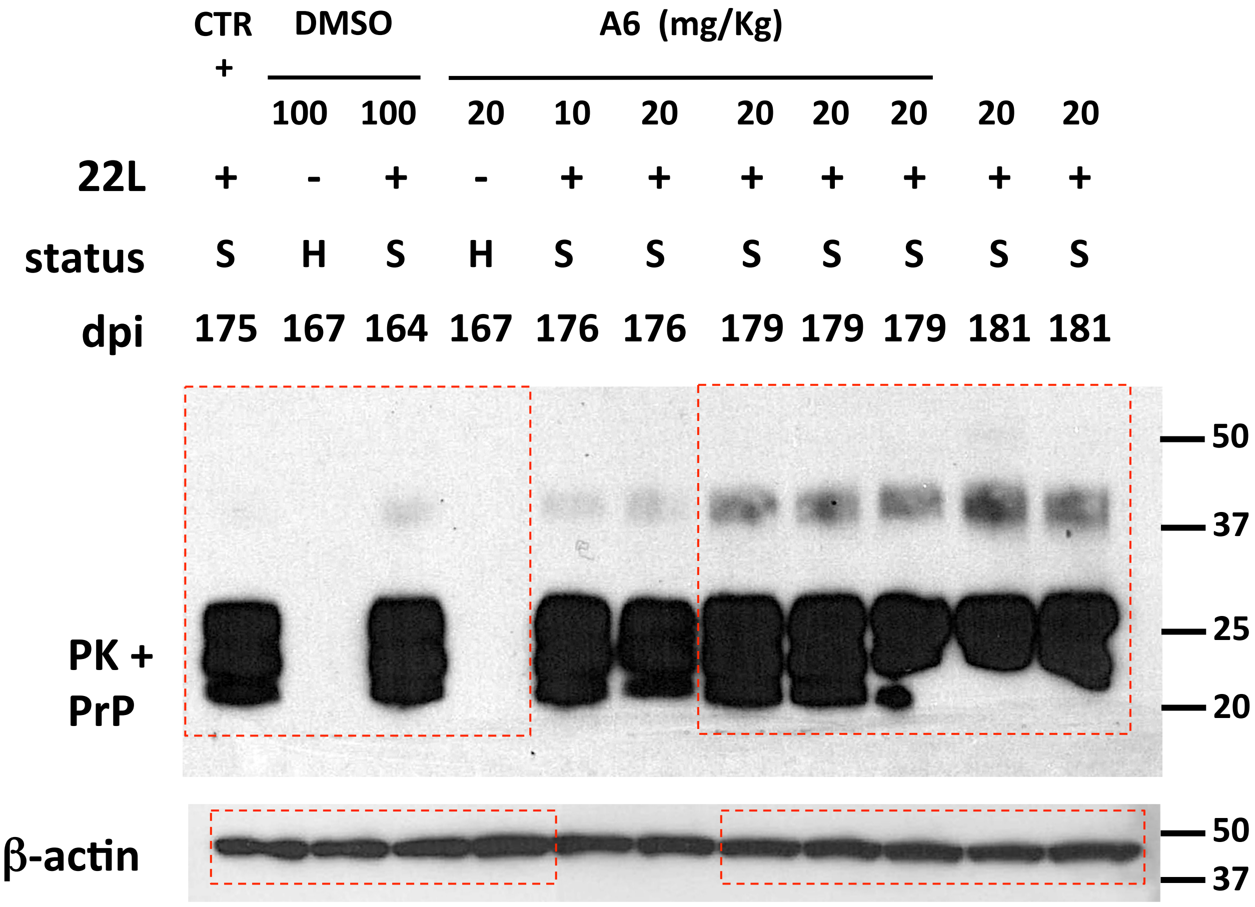

Supplementary Figure S7. Full-length blots presented in Figure 5.
