# Supplementary material for: Low doses of bioherbicide favour prion aggregation and propagation in vivo
Source: Sci Rep. 2018 May 23;8:8023. doi: 10.1038/s41598-018-25966-9 (PMC5966510; doi:10.1038/s41598-018-25966-9)
Supplement: Supplementary file 2 — Original blots [file 41598_2018_25966_MOESM2_ESM.zip › Original blots.pdf]

Title : Low doses of a bioherbicide favours prion aggregation and propagation in vivo

Authors : Pierre-André Lafon, Thibaut Imberdis, Yunyun Wang, Joan Torrent, Mike Robitzer, Elisabeth Huetter, Maria-Teresa Alvarez-Martinez, Nathalie Chevallier, Laurent Givalois, Catherine Desrumaux , Jianfeng Liu and Véronique Perrier.

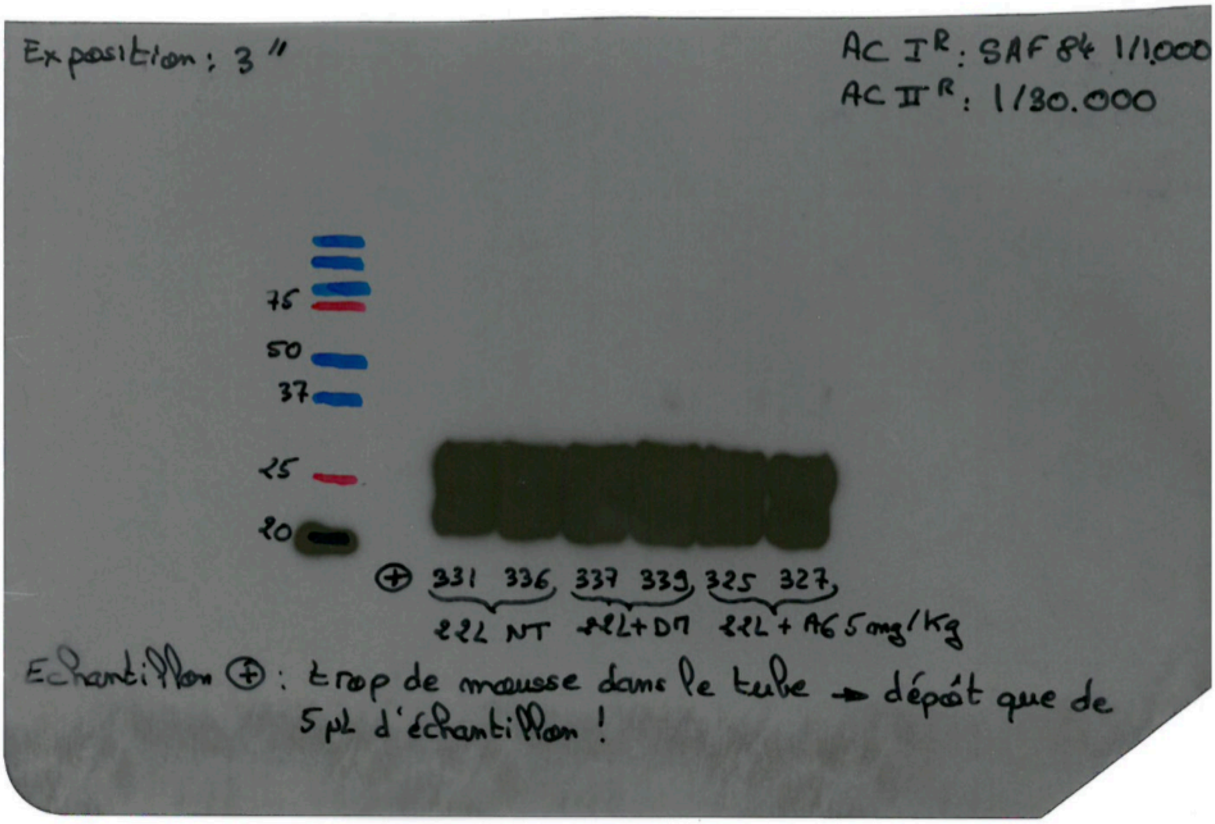
Exposition : 3"
AC I R : SAF 84 1/1.000
AC II R : 1/30.000
75
50
37
25
20
⊕ 331 336 337 339 325 327
22L NT 22L+D7 22L + A6 5mg/Kg
Echantillon ⊕ : trop de mousse dans le tube → dépôt que de 5µL d'échantillon !

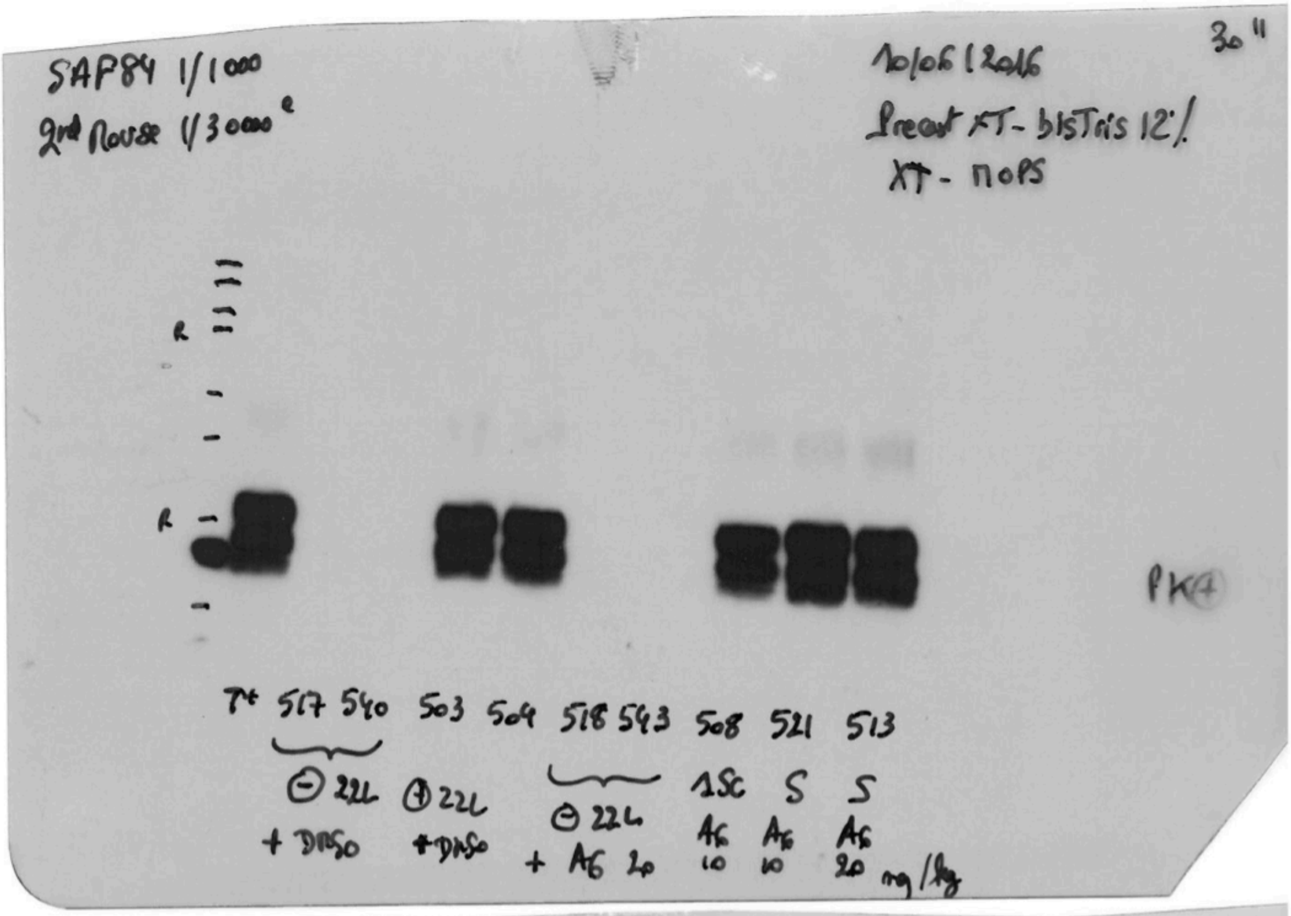
SAP84 1/1000
2nd mouse 1/30000e
10/06/2016
30"
Precast XT-bisTris 12%
XT-MOPS
PK+
T+ 517 540 503 504 518 543 508 521 513
⊖ 22L + DMSO
⊕ 22L + DMSO
⊖ 22L + AG 20
1Sc AG 10
S AG 10
S AG 20
mg/kg

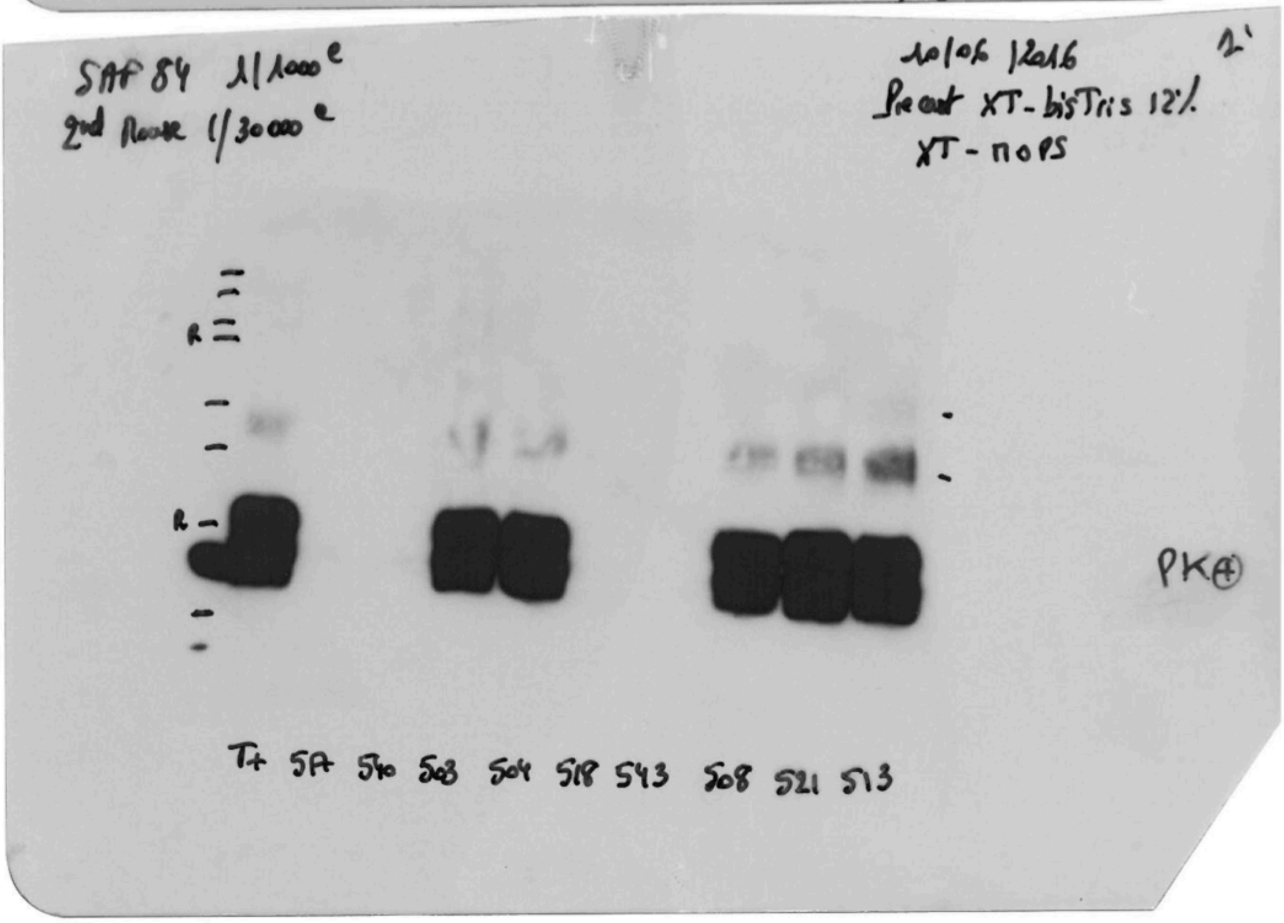
SAP84 1/1000e
2nd mouse 1/30000e
10/06/2016
2'
Precast XT-bisTris 12%
XT-MOPS
PK+
T+ 517 540 503 504 518 543 508 521 513

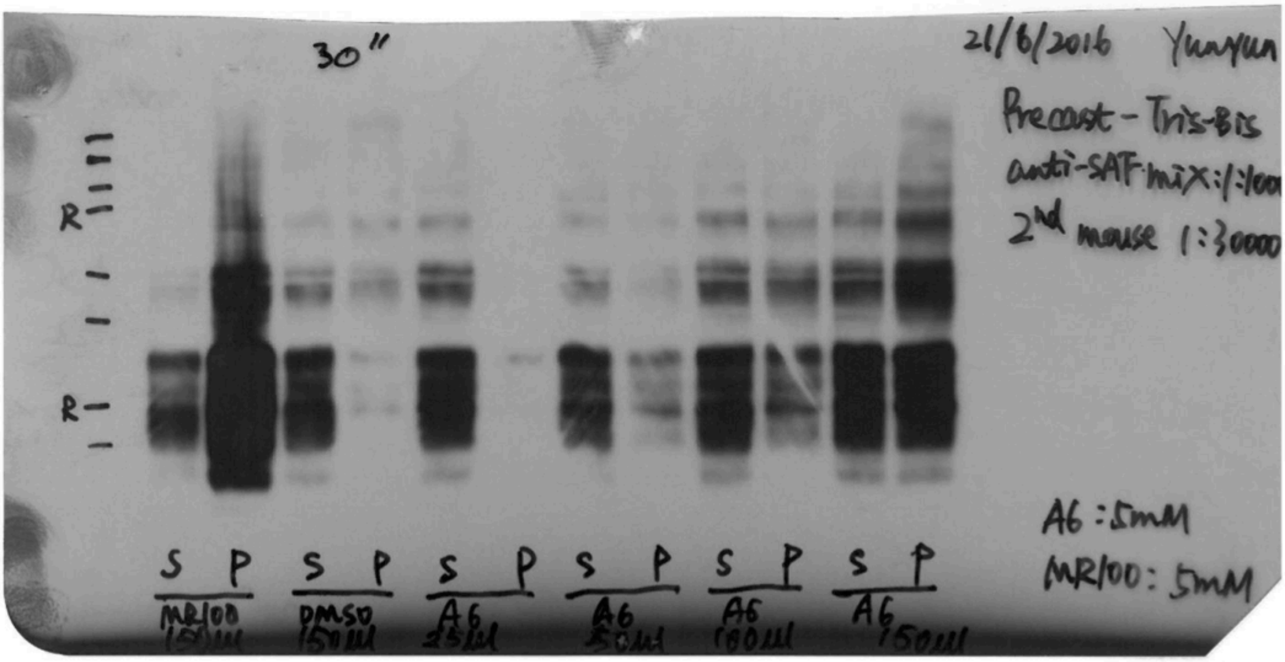
30"
21/6/2016 Yunyun
Precast - Tris-Bis
anti-SAF mix 1:1000
2nd mouse 1:30000
R
R
S P S P S P S P S P S P
MR100 150μl
DMSO 150μl
A6 25μl
A6 50μl
A6 100μl
A6 150μl
A6: 5mM
MR100: 5mM

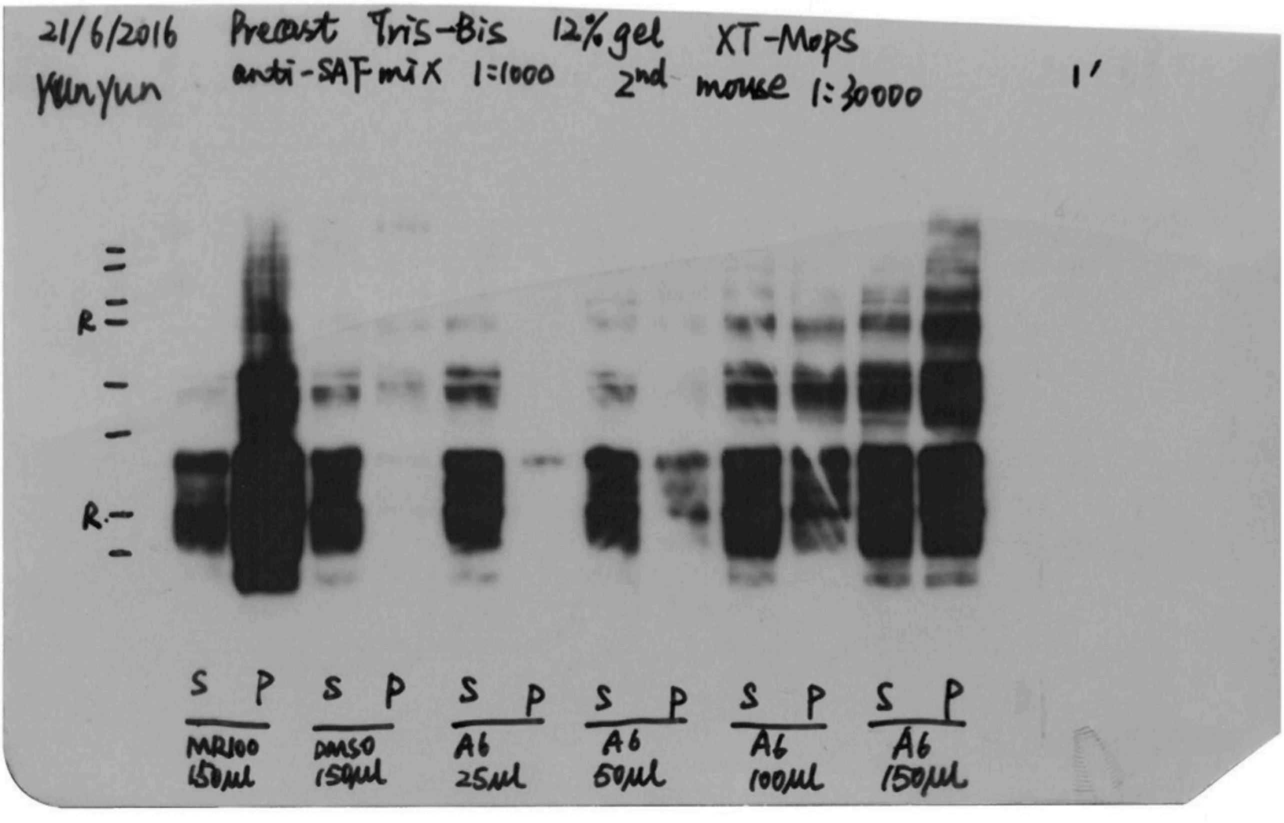
21/6/2016 Precast Tris-Bis 12% gel XT-Mops
Yunyun anti-SAF mix 1:1000 2nd mouse 1:30000
1'
R
R
S P S P S P S P S P S P
MR100 150μl
DMSO 150μl
A6 25μl
A6 50μl
A6 100μl
A6 150μl

SAF mix : 1/1000ème ON à 4°C [28/11/17]

AC II R : 1/2000ème

Test RCA gamme A6 avec homogénat de cerveau WT (NBH)

Temps exposition : 10 min

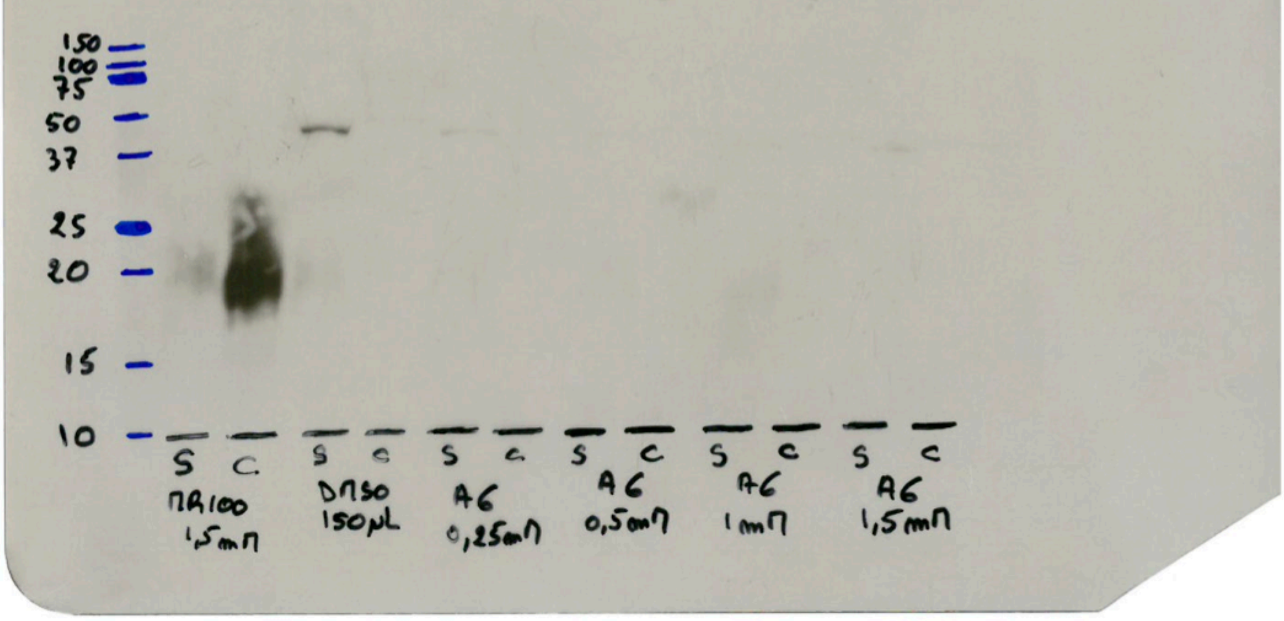

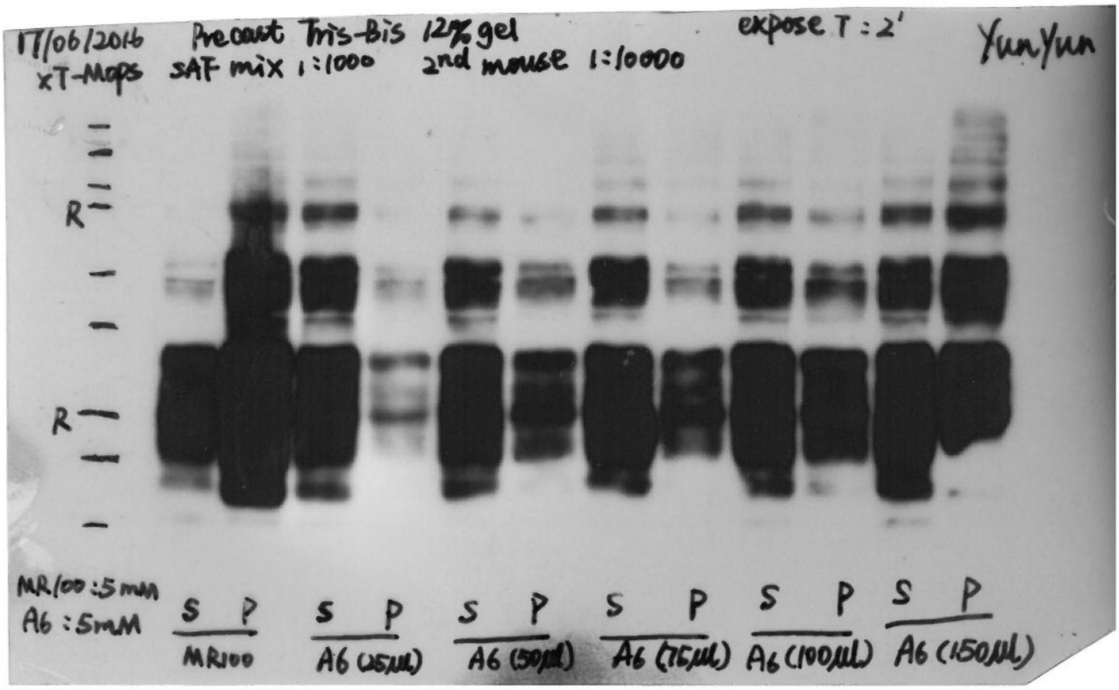
17/06/2016 Precast Tris-Bis 12% gel
XT-Mops SAF mix 1:1000 2nd mouse 1:10000
expose T: 2'
YunYun
R
R
MR100: 5mM
A6: 5mM
S P S P S P S P S P S P
MR100
A6 (25μL)
A6 (50μl)
A6 (75μL)
A6 (100μL)
A6 (150μL)
